# N6-methyladenosine-related non-coding RNAs are potential prognostic and immunotherapeutic responsiveness biomarkers for bladder cancer

**DOI:** 10.1007/s13167-021-00259-w

**Published:** 2021-10-21

**Authors:** Miaolong Lu, Hailun Zhan, Bolong Liu, Dongyang Li, Wenbiao Li, Xuelian Chen, Xiangfu Zhou

**Affiliations:** Department of Urology, The Third Affiliated Hospital of Sun Yat-Sen University, 600 W Tianhe Rd, Guangzhou, Guangdong 510630 People’s Republic of China

**Keywords:** Bladder cancer, N6-methyladenosine (m^6^A) modification, Non-coding RNAs (ncRNAs), Prognostic biomarkers, Immunotherapy responsiveness, Predictive preventive personalized medicine (PPPM)

## Abstract

**Background:**

Bladder cancer (BC) is a commonly occurring malignant tumor of the urinary system, demonstrating high global morbidity and mortality rates. BC currently lacks widely accepted biomarkers and its predictive, preventive, and personalized medicine (PPPM) is still unsatisfactory. N6-methyladenosine (m^6^A) modification and non-coding RNAs (ncRNAs) have been shown to be effective prognostic and immunotherapeutic responsiveness biomarkers and contribute to PPPM for various tumors. However, their role in BC remains unclear.

**Methods:**

m^6^A-related ncRNAs (lncRNAs and miRNAs) were identified through a comprehensive analysis of TCGA, starBase, and m6A2Target databases. Using TCGA dataset (training set), univariate and least absolute shrinkage and selection operator (LASSO) regression analyses were performed to develop an m^6^A-related ncRNA–based prognostic risk model. Kaplan-Meier analysis of overall survival (OS) and receiver operating characteristic (ROC) curves were used to verify the prognostic evaluation power of the risk model in the GSE154261 dataset (testing set) from Gene Expression Omnibus (GEO). A nomogram containing independent prognostic factors was developed. Differences in BC clinical characteristics, m^6^A regulators, m^6^A-related ncRNAs, gene expression patterns, and differentially expressed genes (DEGs)–associated molecular networks between the high- and low-risk groups in TCGA dataset were also analyzed. Additionally, the potential applicability of the risk model in the prediction of immunotherapeutic responsiveness was evaluated based on the “IMvigor210CoreBiologies” data set.

**Results:**

We identified 183 m^6^A-related ncRNAs, of which 14 were related to OS. LASSO regression analysis was further used to develop a prognostic risk model that included 10 m^6^A-related ncRNAs (BAALC-AS1, MIR324, MIR191, MIR25, AC023509.1, AL021707.1, AC026362.1, GATA2-AS1, AC012065.2, and HCP5). The risk model showed an excellent prognostic evaluation performance in both TCGA and GSE154261 datasets, with ROC curve areas under the curve (AUC) of 0.62 and 0.83, respectively. A nomogram containing 3 independent prognostic factors (risk score, age, and clinical stage) was developed and was found to demonstrate high prognostic prediction accuracy (AUC = 0.83). Moreover, the risk model could also predict BC progression. A higher risk score indicated a higher pathological grade and clinical stage. We identified 1058 DEGs between the high- and low-risk groups in TCGA dataset; these DEGs were involved in 3 molecular network systems, i.e., cellular immune response, cell adhesion, and cellular biological metabolism. Furthermore, the expression levels of 8 m^6^A regulators and 12 m^6^A-related ncRNAs were significantly different between the two groups. Finally, this risk model could be used to predict immunotherapeutic responses.

**Conclusion:**

Our study is the first to explore the potential application value of m^6^A-related ncRNAs in BC. The m^6^A-related ncRNA–based risk model demonstrated excellent performance in predicting prognosis and immunotherapeutic responsiveness. Based on this model, in addition to identifying high-risk patients early to provide them with focused attention and targeted prevention, we can also select beneficiaries of immunotherapy to deliver personalized medical services. Furthermore, the m^6^A-related ncRNAs could elucidate the molecular mechanisms of BC and lead to a new direction for the improvement of PPPM for BC.

**Supplementary Information:**

The online version contains supplementary material available at 10.1007/s13167-021-00259-w.

## Introduction

Bladder cancer (BC) is a malignant tumor of the urinary system and the tenth most commonly reported cancer worldwide, demonstrating high morbidity and mortality rates [1]. BC is classified into two types depending on the existence of tumor invasion into the muscle layer of the bladder, that is, non-muscle-invasive and muscle-invasive BC types are reported. Non-muscle-invasive BC (NMIBC) accounts for approximately 70% of all newly diagnosed BC cases [2]. Transurethral resection of bladder cancer (TURBT) is the first choice of treatment, followed by intravesical bacille Calmette-Guerin (BCG) installations or chemotherapy [3]. Although the 5-year survival rate of NMIBC is approximately 90%, the postoperative recurrence rate is relatively high (50–70%) [4]. Muscle-invasive BC (MIBC) represents approximately 20% of all newly diagnosed BC cases, and exhibits a 5-year survival rate of only 50% after radical cystectomy (RC) and pelvic lymph node dissection, with or without chemotherapy and radiation therapy [2]. Although current comprehensive treatment programs such as surgery, radiotherapy, chemotherapy, and targeted therapy can help prolong the overall survival of patients to a certain extent, the overall recurrence and mortality rates of BC remain high, and the prognosis is poor [3]. As BC is highly heterogeneous, predictive, preventive, personalized medicine (PPPM) is an effective strategy used to improve treatment outcomes and patient prognosis [5]. PPPM requires the use of various effective molecular biomarkers, including early diagnostic and prognostic biomarkers that can help clinicians to identify patients in need of early, aggressive management, and predictive biomarkers that can forecast and stratify responses to emerging targeted therapies [5]. In recent years, with the application of multi-omic approaches in cancer research, many BC biomarkers have been reported [6–8]; however, none of them have been introduced into clinical practice. Therefore, the outcomes of PPPM for BC remain unsatisfactory.

RNA acts as a carrier as it transfers genetic information from DNA to proteins and participates in the regulation of various biological processes [9]. Similar to DNA and proteins, RNA undergoes multiple chemical modifications. Presently, more than 170 RNA modifications have been identified in all living organisms, of which methylation is the most important modification reported [10, 11]. N^6^-methyladenosine (m^6^A) is widely distributed in various types of RNA, including mRNA, tRNA, rRNA, miRNA, and long non-coding RNA (lncRNA) [12, 9]. It is the most commonly documented type of base modification and it is conserved in various eukaryotic organisms [11, 13]. During the process of m^6^A modification, the methyl group on S-adenosylmethionine (SAM) is transferred to the sixth nitrogen atom of adenine; this process is dynamic and reversible and involves three types of regulators, demethylases (erasers), methyltransferases (writers), and methylated binding proteins (readers) [14, 15]. Coupled with the action of m^6^A regulators, m^6^A modifications constitute a rapid mechanism for the coordination of RNA processing and metabolism, and for the regulation of almost all vital normal bioprocesses, including cell differentiation, embryonic development, biological rhythm regulation, heat shock response, and DNA damage repair. [16]. As expected, abnormal m^6^A modifications are closely related to the pathogenesis of multiple diseases, especially tumors [17]. Many studies have shown that abnormal m^6^A modifications, which lead to an imbalance in the expression of oncogenes and tumor suppressor genes, may contribute to the initiation and progression of tumors, and affect patient sensitivity to radiotherapy and chemotherapy, as well as clinical prognosis [10, 18–20].

Although non-coding RNAs (ncRNAs) do not encode proteins, they are considered important regulators of gene expression and are involved in the initiation and development of several diseases [21]. Depending on its length, RNA can be classified into two categories, namely small RNA and long-chain RNA. The former is usually less than 50 nucleotides in length, and includes tRNA, rRNA, and miRNA, while the latter is usually more than 200 nucleotides in length [22]. ncRNAs can regulate gene expression in a variety of ways and play pivotal roles in cancer development and progression [22, 23]. For example, dysregulated ncRNAs possess the potential to initiate tumorigenesis, to promote invasion and metastasis, and to confer drug resistance in BC [24]. Of note, studies have demonstrated that m^6^A modification can cause ncRNA function abnormalities by regulating ncRNA processing, thereby promoting tumorigenesis [25]. For example, the methylation-binding protein, YTHDF3, can recognize the m^6^A site on lncRNA GAS5 and promote its degradation, thus activating the Hippo-YAP signaling pathway which promotes colon cancer promotion [26]. The methyltransferase, METL3, promotes the proliferation of BC cells by accelerating pri-miR221/222 maturation in an m^6^A modification-dependent manner [27]. Currently, only a few studies are available which have explored the role m^6^A ncRNA modifications in the progression of BC, and the molecular mechanisms underlying m^6^A modification in BC have not been comprehensively clarified. Therefore, understanding the role of m^6^A ncRNA modification in BC will help clarify the complex molecular mechanisms underlying BC and aid the identification of biomarkers for early diagnosis and prognostic evaluation.

In the present study, we had explored the potential application value of m^6^A-related ncRNAs in BC. Our results indicate that m^6^A-related ncRNAs may be considered novel biomarkers for predicting BC outcome and immunotherapeutic responsiveness, facilitating patients by providing them with targeted prevention and personalized medical service, thereby contributing to the improvement of PPPM for BC.

## Materials and methods

### Download and preprocessing of relevant data

A total of 408 BC patients from the TCGA database (training set) (https://tcga-data.nci.nih.gov/), with complete survival information, were selected for the present study (Supplementary Fig. 1). Their corresponding RNA-seq (19577 genes) (level 3 read counts) and miRNA-seq (1448 miRNAs) data were obtained from the TCGA database on December 25, 2020. Since the sequenced data generated by using the IlluminaHiSeq_RNASeq and IlluminaHiSeq_miRNASeq sequencing platforms were publicly available, further approval by an ethics committee was not required. This study complied with the publication guidelines provided by TCGA (http://cancergenome.nih.gov/publications/publicationguidelines). The lncRNA annotation file for the Genome Reference Consortium Human Build 38 (GRCh38) was obtained from the GENCODE website (https://www.gencodegenes.org/human/) for the annotation of the lncRNAs in TCGA dataset. For this study, 8 lncRNA types were selected, namely “sense overlapping,” “lincRNA,” “3prime overlapping ncRNA,” “processed transcript,” “sense intronic,” “bidirectional promoter lncRNA,” “non coding,” and “antisense.” By identifying the Ensemble IDs of the genes, 14,068 lncRNAs were identified in the TCGA dataset. The GSE154261 dataset, which was considered the testing set and contained 73 BC samples, was downloaded from the Gene Expression Omnibus (GEO) database (https://ftp.ncbi.nlm.nih.gov/geo/series/GSE154nnn/GSE154261/matrix/). BC immunotherapy data were derived from the “IMvigor210CoreBiologies” R package (http://research-pub.gene.com/IMvigor210CoreBiologies/packageVersions/)[28]. Data on the targeted ncRNA m^6^A regulators were downloaded from the m^6^A2Target database (http://m6a2target.canceromics.org/). Data on the ncRNAs showing interactions with m^6^A regulators were obtained from the starBase database (https://bio.tools/starbase).

### Identification of m^6^A regulators and m^6^A-related ncRNAs

Through an exploration of published literature [29, 30], we identified 20 widely accepted m^6^A regulators, including 6 methyltransferases (METTL3, METTL14, METTL16, VIRMA, ZCCHC4, and WTAP), 2 demethylases (FTO and ALKBH5), and 12 methylated binding proteins (IGF2BP1, IGF2BP2, IGF2BP3, YTHDC1, YHTDF2, YTHDF1, YTHDF2, YTHDF3, HNRNPA2B1, KIAA1429, HNRNPC, and HNRNPG). Pearson’s correlation analysis was performed to initially screen for m^6^A-related ncRNAs (|Pearson’s *r*| > 0.2 and *P* < 0.001) in the TCGA database. m^6^A-related ncRNAs were identified through the following steps: (1) the target ncRNAs of m^6^A regulators in the m^6^A2Target database were identified; (2) data on the ncRNAs exhibiting interactions with m^6^A regulators were obtained from the starBase database; (3) ncRNAs showing overlaps in the results obtained by the above-mentioned method and those obtained via the Pearson correlation analysis were selected; finally, we obtained 183 m^6^A-related ncRNAs.

### Construction and validation of the m^6^A-related ncRNA–based risk score model

Based on the TCGA dataset, through univariate Cox regression analysis, we identified 14 m^6^A-related prognostic ncRNAs. The R package “glmnet” [31] was used to perform least absolute shrinkage and selection operator (LASSO) Cox regression analysis (with the penalty parameter estimated by 10-fold cross-validation), and finally, we established a risk model for BC with 10 m^6^A-related ncRNAs. Risk score was calculated using the following formula:

$$Risk\ score={e}^{\sum_{i=1}^n{a}_i{e}_i}$$

where *n* represents the number of m^6^A-related ncRNAs screened by LASSO, and *a* and *e* represent the coefficients correlated with survival and m^6^A-related ncRNA expression, respectively, as determined via LASSO.

To further evaluate the prognostic performance of this risk model, the GSE154261 dataset obtained from the GEO database was used as the testing set. Using the risk score formula, risk scores were calculated for each patient in the GSE154261 dataset, and patients were divided into high- and low-risk groups based on the median value of the prognostic risk score. The R packages “survMiner” and “survival” were used to perform Kaplan-Meier survival analysis to identify differences in OS between the two groups. The R package “pROC” was used to generate the receiver operating characteristic (ROC) curve and to calculate the area under the curve (AUC) [32].

### Construction and verification of the predictive nomogram

Univariate and multivariate Cox regression analyses were performed to identify independent variables, such as age at diagnosis, sex, race, pathological grade, risk score, and clinical stage. Furthermore, to individualize the predicted 1- and 2-year survival probabilities, the R package “rms” was used to generate a nomogram that included significant clinical characteristics and calibration plots. Correction curves based on the Hosmer-Lemeshow test were generated to compare prediction accuracy between the observed and model-predicted outcomes.

### Functional enrichment analysis of differentially expressed genes (DEGs)

DEGs between the high- and low-risk groups were identified based on the conditions, | log_2_ (fold change)| > 1 and FDR < 0.05, using the R package “DESeq2” [33]. DAVID (version 6.8, https://david.ncifcrf.gov/) was used to perform Gene Ontology (GO) and Kyoto Encyclopedia of Genes and Genomes (KEGG) enrichment and cluster analyses [34].

### Evaluation of the immunotherapeutic responsiveness predictive ability of the risk model

We calculated the risk scores of 348 patients in the “IMvigor210CoreBiologies” dataset. Based on the median values, patients were divided into high- and low-risk groups. Survival analysis was performed to identify differences in the survival status between the two groups. Additionally, we assessed the relationship between risk score and immunotherapeutic responsiveness.

## Results

### Identification of m^6^A-related ncRNAs in BC patients

**Fig. 1:**
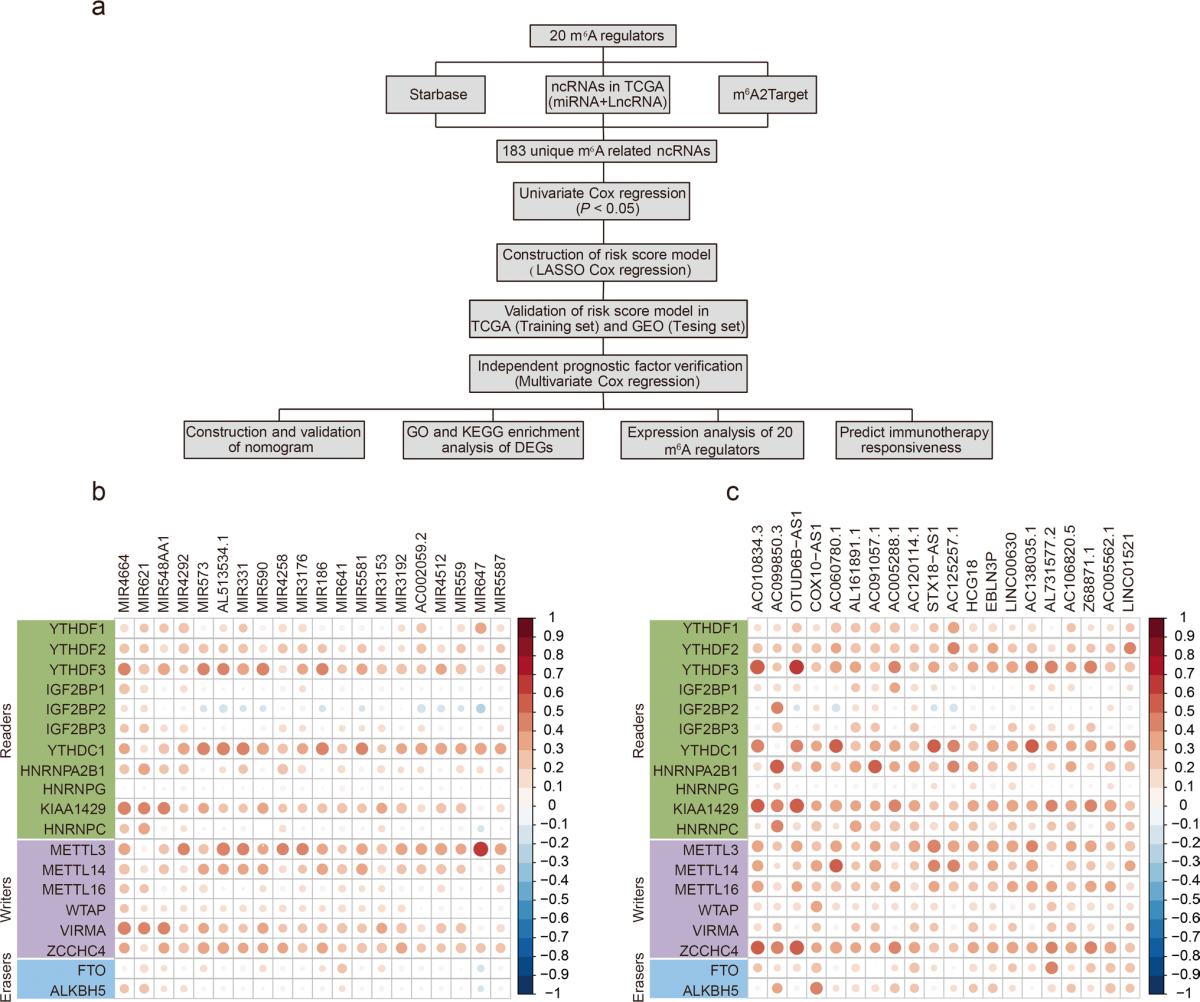
Flowchart and the correlation analysis between ncRNAs (miRNA and lncRNA) and m^6^A regulators in TCGA. **a** Flowchart of this study. **b** Heatmap for the top 20 miRNAs that were most related to m^6^A regulators in TCGA. **c** Heatmap for the top 20 lncRNAs that were most related to m^6^A regulators in TCGA. DEGs: Differentially expressed genes

The study flowchart is illustrated in Fig. 1a. First, we identified m^6^A-related ncRNAs by comprehensively analyzing the results of two databases (starBase and m^6^A2Target) and by performing correlation analyses on m^6^A regulators and ncRNAs (miRNAs and lncRNAs) using the TCGA dataset. After performing univariate Cox regression analysis and LASSO regression analysis, an m^6^A-related ncRNA–based risk score model was constructed. We verified the prognostic evaluation performance of this risk score model using TCGA (training set) and GEO (testing set) datasets. Finally, based on the risk model established herein, patients in the TCGA dataset were divided into the high- and low-risk groups. We further analyzed differences in the survival status, m^6^A regulators, gene expression patterns, and immunotherapeutic responsiveness between the two groups.

**Fig. 2:**
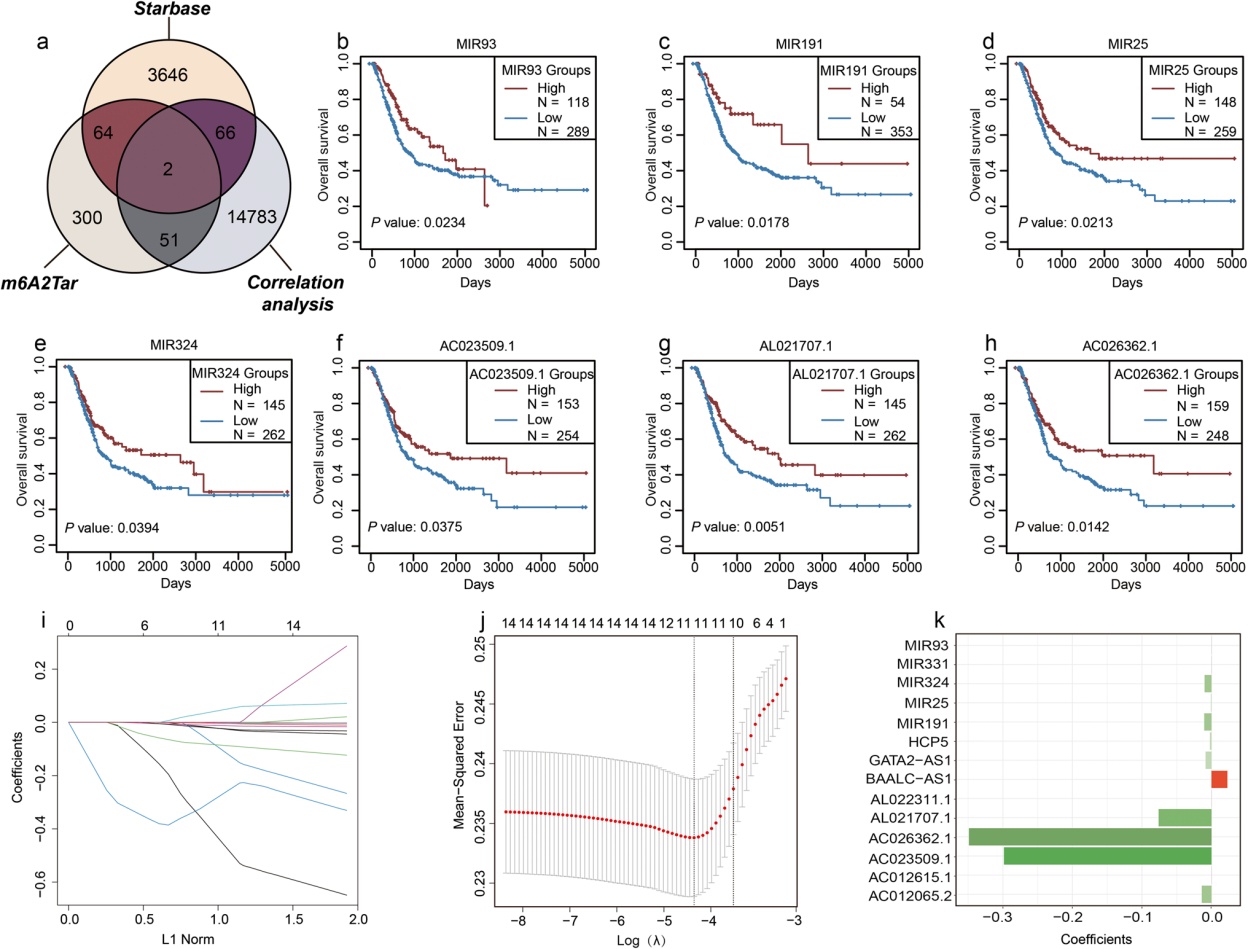
Construction of an m^6^A-related ncRNA–based risk model for BC. **a** 183 m^6^A-related ncRNAs were identified by taking the intersection of starBase, m^6^A2Target, and correlation analysis between 20 m^6^A regulators and ncRNAs in TCGA. **b–h** Kaplan-Meier survival curves of the OS of 7 m^6^A-related ncRNAs. **i** LASSO coefficient profile of 14 OS-related ncRNAs and perpendicular imaginary line were drawn at the value chosen by 10-fold cross-validation. **j** The tuning parameters (log λ) of OS-related ncRNAs were selected to cross-verify the error curve. According to the minimal criterion and 1-se criterion, perpendicular imaginary lines were drawn at the optimal value. **k** Histogram for the regression coefficient of each ncRNA in the risk model

Through Pearson’s correlation analysis of ncRNAs and m^6^A regulators in the TCGA dataset, we obtained 13,596 lncRNAs and 1306 miRNAs that satisfied the filtering criteria. Figure 1b and 1c depict the top 20 miRNAs and lncRNAs, respectively, that were most significantly associated with m^6^A regulators in the TCGA dataset. Overall, lncRNAs were more significantly correlated with m^6^A regulators than miRNAs. Among them, MIR647 and lncRNA OTUD6B-AS1 were found to be significantly correlated with m^6^A regulators METTL3 and YTHDF3, with Pearson’s correlation coefficients of 0.60 (*P* < 0.001) and 0.61 (*P* < 0.001), respectively. Detailed results of the correlation analysis between ncRNAs and m^6^A regulators are listed in Supplementary Tables 1 and 2. Using the m^6^A2Target database, a total of 704 potential downstream m^6^A regulator target genes were identified, including 417 ncRNAs. Using the starBase database, we identified 3778 ncRNAs that established interactions with m^6^A regulators. Then, ncRNAs obtained from three datasets (i.e., the m^6^A regulator and ncRNA correlation analysis, starBase, and m^6^A2Target databases) were investigated and they were found to overlap, and 183 unique m^6^A-related ncRNAs were identified (Fig. 2a).

### Construction and validation of the m^6^A-related ncRNA–based risk model using TCGA dataset

Through univariate Cox regression analysis, we identified 14 m^6^A-related ncRNAs (MIR324, MIR93, MIR331, MIR191, MIR25, AL022311.1, AC023509.1, AC012615.1, AL021707.1, AC026362.1, BAALC-AS1, GATA2-AS1, AC012065.2, and HCP5) which were significantly correlated with OS in TCGA dataset. Among them, 6 m^6^A-related ncRNAs (MIR324, MIR25, AL022311.1, AC012615.1, AC026362.1, and GATA2-AS1) were significantly upregulated in tumor tissues (Supplementary Fig. 2). Survival analysis revealed that the prognosis of BC patients with high expression levels of 7 m^6^A-related ncRNAs (MIR324, MIR93, MIR191, MIR25, AC023509.1, AL021707.1, and AC026362.1) was better than that of patients with low expression levels of these ncRNAs (Fig. 2b–h). Through LASSO regression analysis of 14 OS-related ncRNAs obtained via univariate Cox regression analysis performed to eliminate redundant factors, we established the prognostic risk score formula as follows:

Risk score = (BAALC-AS1 *0.023) − (MIR324 *0.01) − (MIR191 *0.01) − (MIR25 *0.001) − (AC023509.1 *0.3) − (AL021707.1 *0.08) − (AC026362.1 *0.35) − (GATA2-AS1 *0.01) − (AC012065.2 *0.013) − (HCP5 *0.002) (Fig. 2i–k).

**Fig. 3:**
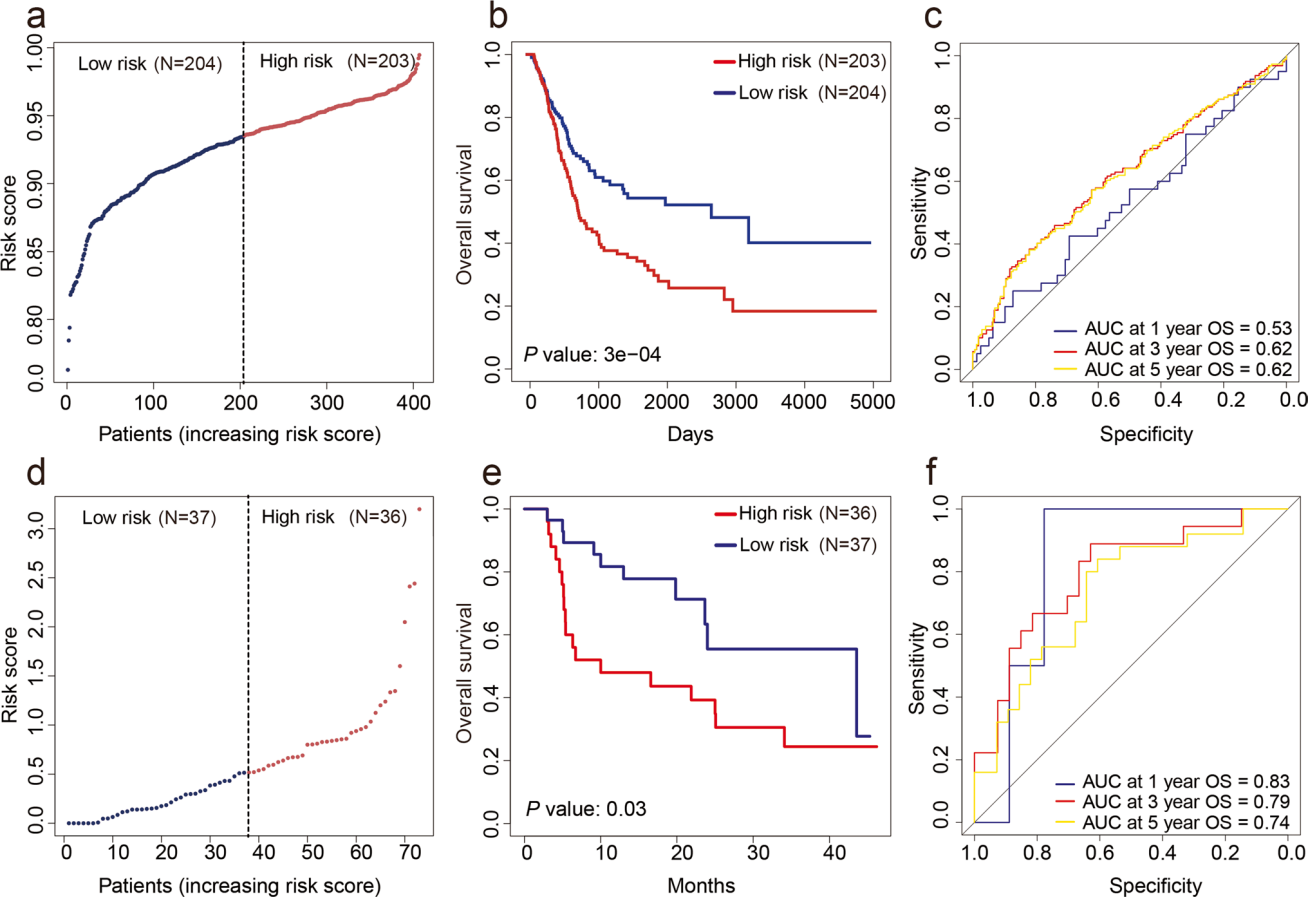
Validation of the prognostic evaluation power of m^6^A-related ncRNA–based risk model in TCGA and GSE154261. **a** Distribution of m^6^A-related ncRNAs based risk score for TCGA. **b** Kaplan-Meier survival curves of the OS of patients in the high- and low-risk groups for TCGA. **c** ROC analysis for predicting the probability of OS at 1, 3, and 5 years for TCGA. **d** Distribution of m^6^A-related ncRNAs based risk score for GSE154261. **e** Kaplan-Meier survival curves of the OS of patients in the high- and low-risk groups for GSE154261. **f** ROC analysis for predicting the probability of OS at 1, 3, and 5 years for GSE154261

Next, we assessed the prognostic evaluation power of the risk model in the TCGA dataset. Based on the median value of the risk score (0.934), samples were divided into high-risk (*n* = 203) and low-risk (*n* = 204) groups. Figure 3a depicts the risk score distribution between the patients. Survival analysis showed that there were significant differences in the survival status between the two groups (*P* < 0.01), and the survival time of the low-risk group was longer than that of the high-risk group (Fig. 3b). Assessment of the predictive power of the risk score on survival showed that the maximum AUC of the ROC curve was 0.62 (Fig. 3c). Additionally, ROC analysis revealed that the risk model exhibited a good predictive power for the probability of OS at 3 and 5 years for the TCGA dataset (Fig. 3c).

### Validation of the prognostic evaluation power of the m^6^A-related ncRNA–based risk model using the testing set

To further explore the prognostic evaluation efficiency of this risk model, we adopted the same method as described above to analyze the GSE154261 dataset (testing set) obtained from the GEO database. Figure 3d depicts the risk score distribution of 73 samples in the GSE154261 dataset. Based on the median value of the risk score (0.514), the samples were divided into high-risk (*n* = 36) and low-risk (*n* = 37) groups. As shown in Fig. 3e, there was a significant difference in survival time between the two groups, with the low-risk group exhibiting a longer survival time than the high-risk group (*P* = 0.03). This finding was consistent with that of the survival analysis performed using the TCGA dataset. Assessment of the predictive power of the risk score on survival showed that the maximum AUC of the ROC curve was 0.83 (Fig. 3f). The risk model showed excellent predictive power for the probability of OS at 1, 3, and 5 years (Fig. 3f).

### The m^6^A-related ncRNA–based risk model is an independent prognostic factor for BC

**Fig. 4:**
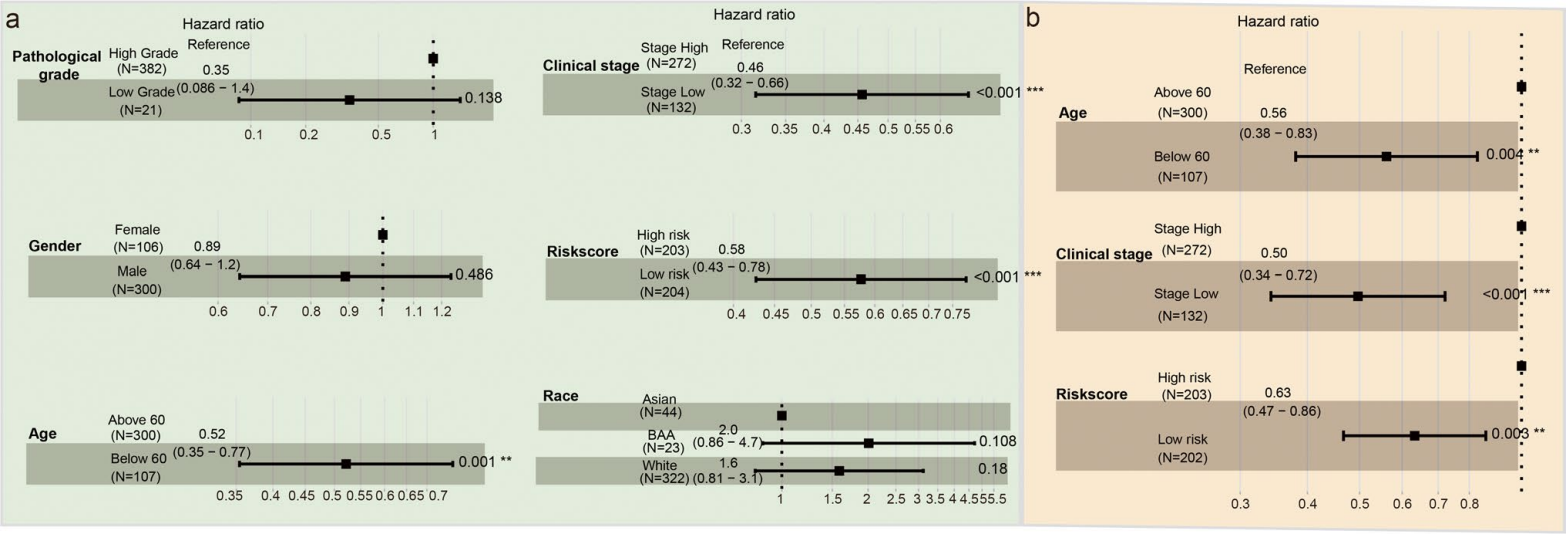
m^6^A-related ncRNA–based risk model was an independent prognostic factor for BC. **a** Univariate cox regression analysis including age, gender, pathological grade, race, clinical stage, and risk score. **b** Multivariate cox regression analysis identified 3 independent prognostic factors (age, clinical stage, and risk score). * represent *P* value < 0.05, ** *P* value < 0.01 and *** *P* value < 0.001

To determine whether the risk model was an independent prognostic factor, the risk score was considered as a new variable and we performed univariate and multivariate Cox regression analyses; other clinical characteristics such as age, sex, pathological grade, race, and clinical stage were also considered. The results of the univariate Cox regression analysis showed that the risk score (*P* < 0.001, hazard ratio [HR] = 0.58, 95% CI: 0.43–0.78), as well as age (*P* = 0.001, HR = 0.52, 95% confidence interval [CI]: 0.35–0.77) and clinical stage (*P* < 0.001, HR = 0.46, 95% CI: 0.32–0.66), was significantly correlated with OS (Fig. 4a). Additionally, a higher risk score indicated poorer OS in BC patients. Multivariate Cox regression analysis results further confirmed that a “high risk score” was an independent factor associated with poor OS (*P* = 0.003, HR = 0.63, 95% CI: 0.47–0.86) (Fig. 4b).

### Construction and validation of the prognostic nomogram

**Fig. 5:**
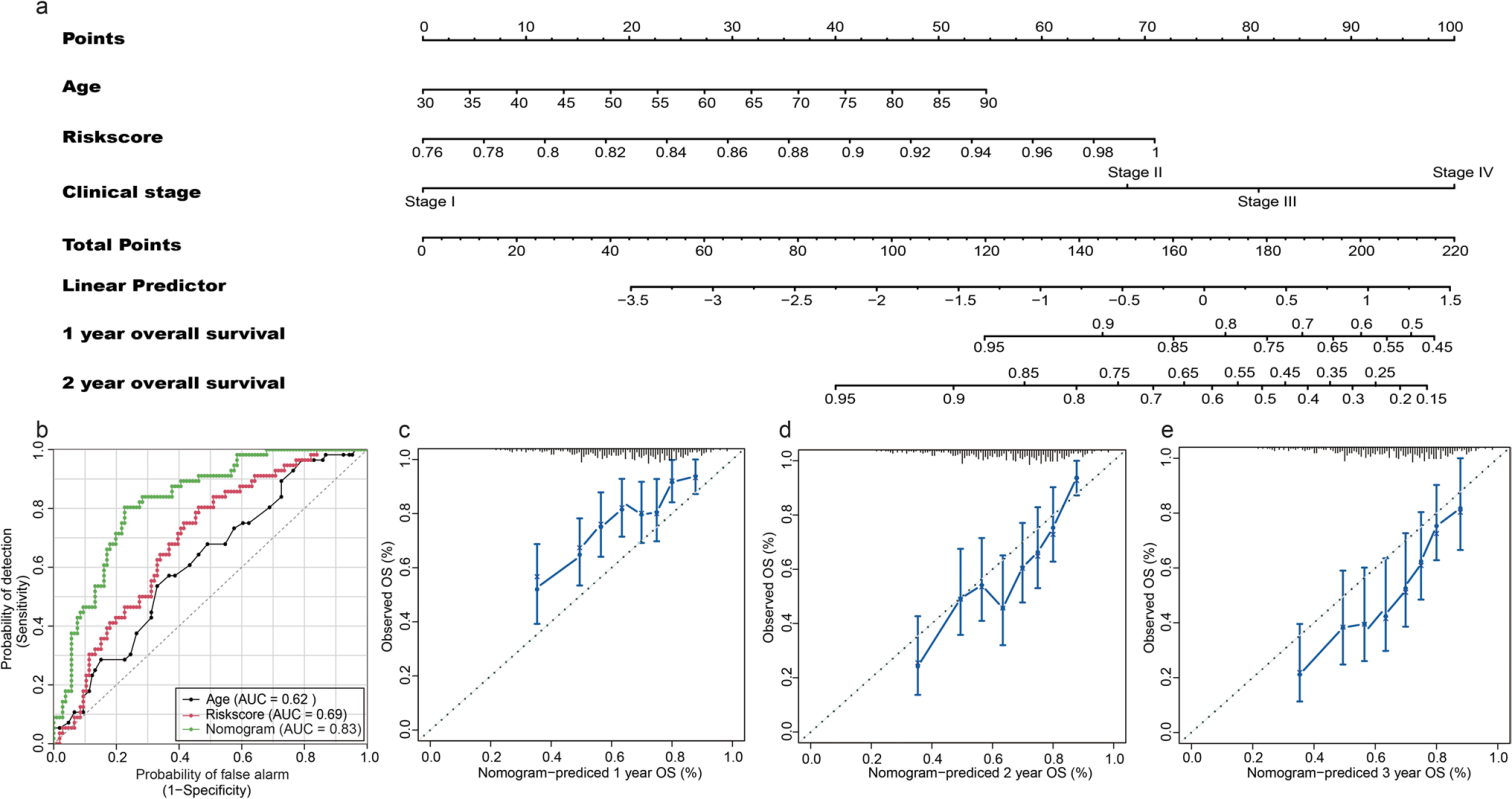
Construction and validation of the prognostic nomogram. **a** Nomogram for predicting the probability of 1- and 2-year OS for BC. **b** ROC curve analyses of age, risk score, and nomogram. **c–e** Calibration plot of the nomogram for predicting the probability of OS at 1, 2, and 3 years

To provide clinicians with a quantitative approach for predicting the prognosis of BC, a nomogram that was constructed by integrating risk score and other independent clinical risk factors (age and clinical stage) was established and used for further analysis. The line segment corresponding to each independent risk factor in the nomogram is marked with a scale, which represents the range of each independent risk factor, and the length of the line segment reflects the contribution of each independent risk factor. The value of each independent risk factor corresponding to the first row of “Points” is the single score. The total score obtained by adding up single scores will eventually correspond to the “Total Points” row. Finally, according to the position of total score at different observation times, OS is predicted. Poor prognosis was represented by a higher total number of points on the nomogram (Fig. 5a). We also compared the predictive accuracy of this nomogram with that of the analysis based on age and risk score and found that the performance of the nomogram (AUC = 0.83) was better than the analysis conducted using data on age (AUC = 0.62) and risk score (AUC = 0.69) (Fig. 5b). Additionally, predictions proposed by using the calibration curve of the nomogram for the first-, second-, and third-year OS were closer to the observed OS (Fig. 5c–e).

### Evaluation of the prognostic risk model and the clinical characteristics of BC

**Fig. 6:**
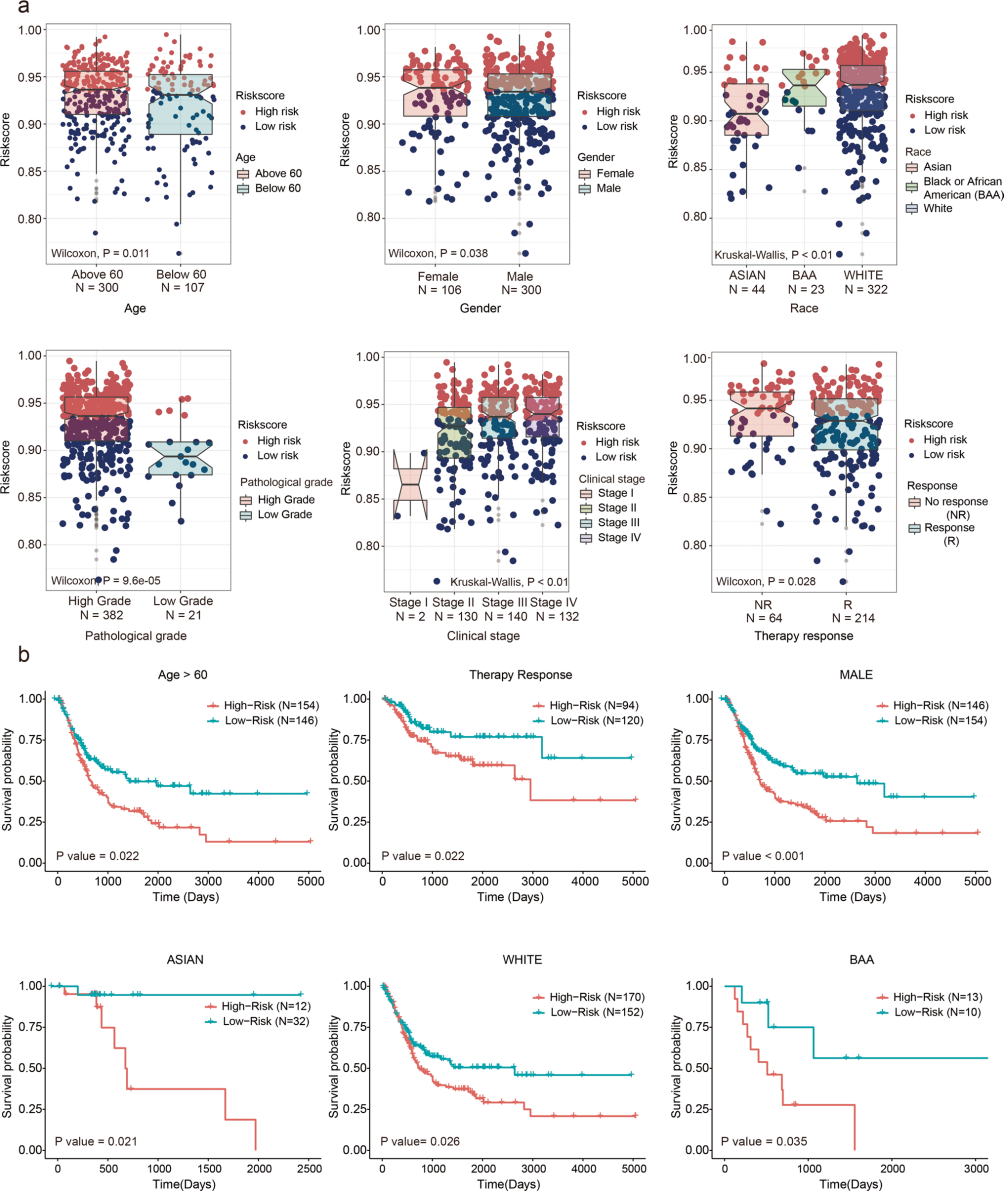
Evaluation of the prognostic risk model and clinical characteristics of BC. **a** Distribution differences of risk score between age, gender, race, pathological grade, clinical stage, and treatment response in TCGA. **b** Kaplan-Meier curves of OS for subgroups with different clinical characteristics in TCGA

Having confirmed that the risk model was an efficient tool for predicting prognosis, we aimed to ascertain if it could reflect the clinical characteristics of BC. To achieve this purpose, we analyzed the correlation between risk score and the clinical characteristics of BC. As shown in Fig. 6a, patients older than 60 years of age presented with higher risk scores than those younger than 60 years; female patients presented with higher risk scores than male patients; Black or African American (BAA) and White patients presented with higher risk scores than Asians; patients with higher BC pathological grades presented with higher risk scores than those with lower BC pathological grades; as the tumor TNM grade increased, the risk score also tended to increase; patients who presented with no initial treatment response (NR) exhibited higher risk scores than those who demonstrated an initial treatment response. These results indicated that the risk model was related to the progression of BC. Subsequently, we conducted a survival analysis for the OS of subgroups with different clinical characteristics. We found that the OS of patients in the low-risk group was better than that of patients in the high-risk group, in the “older than 60 years” group, in the “therapy response” group, in the male patients’ group, and in patients in three races (BAA, White, and Asian) (Fig. 6b).

### Evaluation of molecular network alterations between the high- and low-risk groups

Owing to the significant differences in survival status observed between the high- and low-risk groups, we analyzed the gene expression profiles of the two groups. According to the previously established criteria for DEGs, we obtained data on 1058 DEGs (Supplementary Table 3 and Fig. 3a). As shown in supplementary Fig. 3b, DEGs exhibited significantly different expression patterns between the high- and low-risk groups. To understand the molecular network alterations involved in these DEGs, we performed cluster analysis using the results of the GO and KEGG enrichment analyses. Each cluster represented a type of molecular network system with similar functions during carcinogenesis. We found that the 3 clusters were significantly enriched (Table 1). Cluster 1 was significantly associated with the cellular immune response, which comprised multiple biological processes and pathways related to the activation and chemotaxis of immune cells, such as neutrophil chemotaxis, monocyte chemotaxis, lymphocyte chemotaxis, and cellular response to interferon-gamma and interleukin-1. Cluster 2 was associated with cell adhesion and the interaction between cells and the extracellular matrix (ECM). Cluster 3 was involved in cellular biological metabolism, including the metabolic processes of a variety of important compounds, such as steroids, retinol, linoleic acid, and arachidonic acid. Additionally, the cytochrome P450 pathway, which is involved in drug metabolism, belonged to this cluster.

**Table 1:** Molecular network differences between high- and low-risk groups

**Category**	**Term**	***P*** **value**	**FDR**
**Cluster 1**			
KEGG_PATHWAY	Cytokine-cytokine receptor interaction	2.74E–17	5.75E–15
GOTERM_BP_DIRECT	Chemokine-mediated signaling pathway	1.90E–14	1.64E–11
GOTERM_BP_DIRECT	Neutrophil chemotaxis	2.75E–14	1.90E–11
GOTERM_MF_DIRECT	Chemokine activity	1.60E–13	1.61E–10
GOTERM_BP_DIRECT	Chemotaxis	2.15E–12	1.24E–09
GOTERM_BP_DIRECT	Monocyte chemotaxis	2.17E–11	1.07E–08
GOTERM_BP_DIRECT	Cell-cell signaling	3.28E–11	1.42E–08
GOTERM_BP_DIRECT	Cellular response to interferon-gamma	6.85E–10	2.15E–07
GOTERM_BP_DIRECT	Cellular response to interleukin-1	7.61E–10	2.19E–07
GOTERM_BP_DIRECT	Positive regulation of inflammatory response	1.31E–09	3.48E–07
GOTERM_BP_DIRECT	Cell chemotaxis	4.82E–08	9.27E–06
GOTERM_BP_DIRECT	Lymphocyte chemotaxis	1.14E–07	2.08E–05
GOTERM_BP_DIRECT	Positive regulation of ERK1 and ERK2 cascade	3.02E–07	4.75E–05
GOTERM_BP_DIRECT	Cellular response to tumor necrosis factor	4.67E–07	7.02E–05
GOTERM_MF_DIRECT	CCR chemokine receptor binding	1.44E–05	1.32E–03
KEGG_PATHWAY	Chemokine signaling pathway	4.16E–05	7.93E–04
**Cluster 2**			
KEGG_PATHWAY	ECM-receptor interaction	2.91E–06	6.80E–05
KEGG_PATHWAY	Focal adhesion	1.30E–05	2.73E–04
KEGG_PATHWAY	PI3K-Akt signaling pathway	1.73E–04	2.42E–03
**Cluster 3**			
GOTERM_BP_DIRECT	Steroid metabolic process	8.51E–05	7.55E–03
KEGG_PATHWAY	Chemical carcinogenesis	2.49E–04	3.07E–03
KEGG_PATHWAY	Drug metabolism - cytochrome P450	6.30E–04	6.97E–03
KEGG_PATHWAY	Metabolism of xenobiotics by cytochrome P450	4.33E–03	2.82E–02
KEGG_PATHWAY	Retinol metabolism	4.45E–03	2.82E–02
KEGG_PATHWAY	Linoleic acid metabolism	2.21E–04	2.90E–03
KEGG_PATHWAY	Arachidonic acid metabolism	8.73E–04	8.34E–03

### m^6^A regulator and m^6^A-related ncRNA expression differences between the high- and low-risk groups

**Fig. 7:**
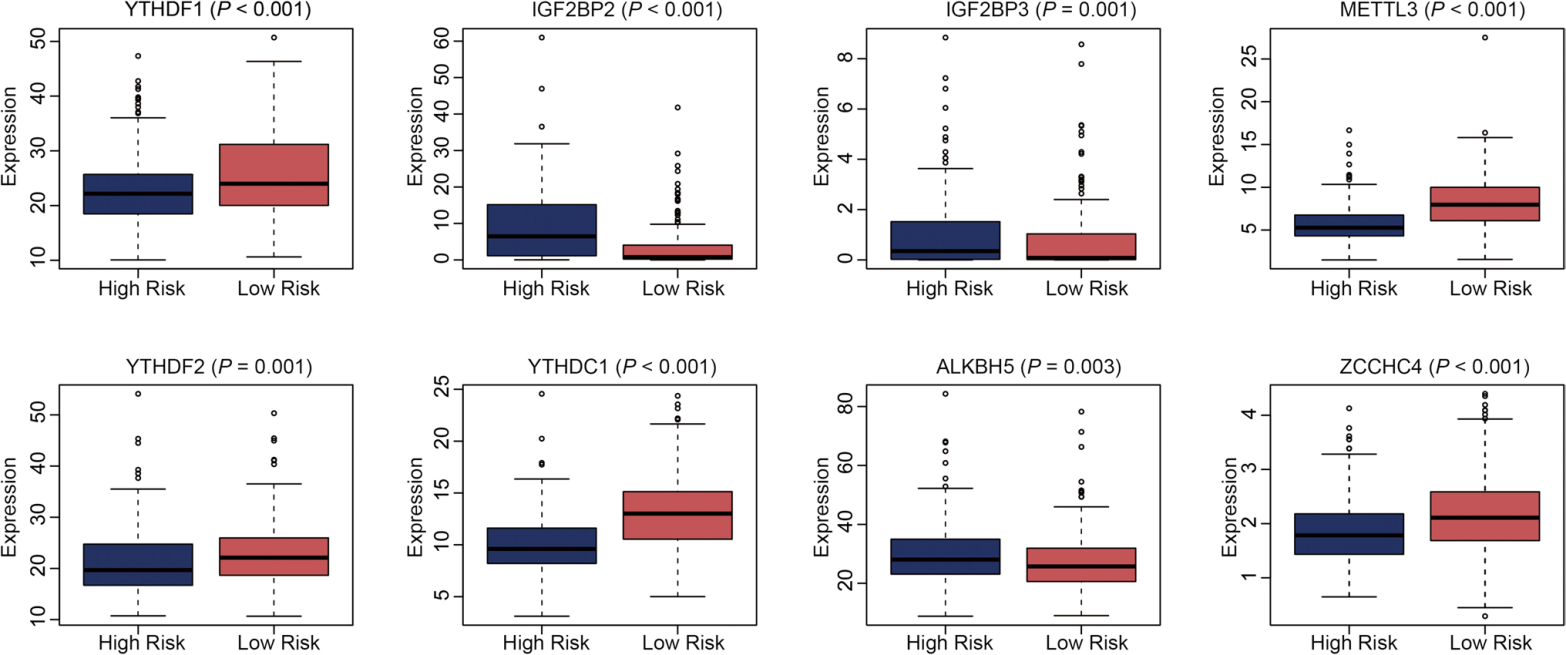
Expression pattern differences of m^6^A regulators between the high- and low-risk groups in TCGA

m^6^A regulators, which are critical molecules in the modification process, also showed expression differences between the high- and low-risk groups. We discovered 8 m^6^A regulators; of these, IGF2BP2, IGF2BP3, and ALKBH5 were highly expressed in the high-risk group, while YTHDF1, METTL3, YTHDF2, YTHDC1, and ZCCHC4 were highly expressed in the low-risk group (Fig. 7). Additionally, among the 14 OS-related ncRNAs, 12 (MIR324, MIR93, MIR331, MIR25, AL022311.1, AC023509.1, AC012615.1, AL021707.1, AC026362.1, GATA2−AS1, AC012065.2, and HCP5) were differentially expressed between the high- and low-risk groups. Apart from HCP5, which was highly expressed in the high-risk group, the other ncRNAs were not highly expressed in this group (Supplementary Fig. 4).

### The risk model may aid the selection of patients who may benefit from immunotherapy

**Fig. 8:**
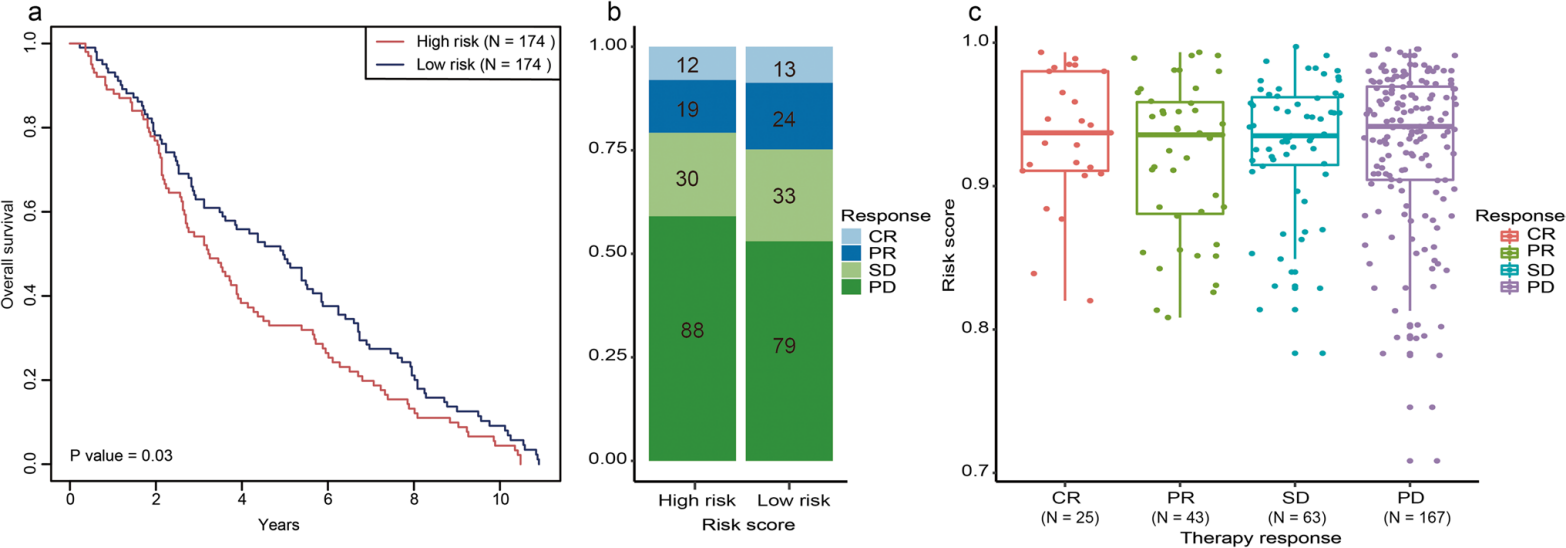
m^6^A-related ncRNA model-based risk model could predict the responsiveness of BC immunotherapy. **a** Kaplan-Meier curves of OS for high- and low-risk groups in “IMvigor210CoreBiologies” set. **b** Differences in immunotherapy response between the high-and low-risk groups. **c** Distribution of risk score in different immunotherapy response groups. CR: complete response, PR: partial response, SD: stable disease, PD: progressive disease

Immunotherapy has revolutionized the treatment of BC; however, not all patients can derive benefits. Screening for potential beneficiaries of BC immunotherapy is an effective means of improving disease prognosis and achieving precise medical treatment for BC. Hence, we evaluated the ability of the risk model to be used to predict BC immunotherapeutic responsiveness. As described previously, the risk score formula was used to calculate the risk score for each sample (*n* = 348) in the BC immunotherapy dataset, and based on the median value of the risk score (0.94), the samples were divided into the high-risk (*n* = 174) and low-risk (*n* = 174) groups. Survival analysis revealed that there were significant differences (*P* = 0.03) in survival status between the two groups, with the survival time of patients in the high-risk group being shorter than that of patients in the low-risk group (Fig. 8a). Concerning immunotherapeutic effects, the high-risk group presented with a lower response rate (RR, the ratio of patient with complete response (CR) and partial response (PR)) than the low-risk group (20.81% vs. 24.83%) (Fig. 8b). The above result indicated that there were differences in immunotherapy responsiveness between the high- and low-risk groups. Furthermore, patients with disease progression (PD) presented with higher risk scores than those observed in CR, PR, or those with stable disease (SD) (Fig. 8c). In summary, our results indicated that this risk model could be a promising biomarker to predict immunotherapeutic responses.

## Discussion

The advent of multi-omics technology has advanced our understanding of the molecular mechanisms of tumorigenesis in a profound manner, and has resulted in changes in the clinical treatment strategies adopted for several tumors [6]. Few molecular biomarkers have been established as an indispensable approach of PPPM. For example, PSA and AFP have been used as conventional biomarkers for the diagnosis of prostate and liver cancer, respectively [35, 36]. EGFR serves as a biomarker for tumor pathological classification and targeted therapy in lung adenocarcinoma [37]. Research conducted in the last 30 years, which has been focused on the molecular mechanisms of BC, has provided us with information on a substantial number of potential molecular biomarkers, including early diagnostic biomarkers (such as TERT promoter mutations and chromatin-modifying gene alterations) [7], prognostic biomarkers that can be used to identify high-risk patients who require active surveillance and early aggressive treatment (such as p53, pRB, p21, p27, cyclins D1 and D3, fibroblast growth factor receptor 3 (FGFR3), and Ki-67) [38], and predictive biomarkers which are used to forecast and stratify the patient’s response to chemotherapy or targeted therapy (such as EGFR, VEGFR, Bcl-2, EMMPRIN, and survivin) [38]. In addition to the above-mentioned BC tissue-based molecular biomarkers, a wide range of potential molecular biomarkers have been discovered and reported in urine [39], circulating tumor cells [40], and exosomes [41]. Unfortunately, due to the lack of availability of sufficient validation and prospective studies, no molecular biomarker has been ratified for use in the clinical management of BC. Presently, pathological grade remains a decisive indicator for evaluating the prognosis of BC. However, in clinical practice, it is not uncommon for patients with the same pathological grade to exhibit significant differences in prognosis. This implies that due to the heterogeneity of BC, the current prognosis assessment system cannot meet the needs of personalized medicine. Therefore, for BC, which demonstrates high recurrence and mortality rates with limited diagnostic and treatment methods, the identification of novel biomarkers to improve PPPM is the need of the hour.

Previous studies have shown that both m^6^A modification and ncRNAs are involved in the initiation and development of a variety of tumors [42], and can therefore be considered as potential molecular biomarkers [16, 22]. Of note, there exists a complex interactive regulation between m^6^A modification and ncRNAs. Studies have shown that m^6^A modification can promote tumor cell proliferation, invasion, and metastasis by regulating the splicing, maturation, transport, and stability of ncRNAs. For example, as a “writer,” METTL3 can not only promote the progression of pancreatic cancer by regulating the maturation of miR-25-3P [43], but it can also promote the tumorigenesis and metastasis of nasopharyngeal carcinoma by enhancing the stability of lncRNA FAM255A [44]. Furthermore, ncRNAs may target m^6^A regulators as competitive endogenous RNAs, thereby affecting tumor progression. For example, low miR-1266 expression promotes colorectal cancer progression by regulating demethylase FTO [45]. LncRNA GAS5-AS1 establishes interaction with demethylase ALKBH5 to increase the stability of GAS5, thereby suppressing the growth and metastasis of cervical cancer [46]. Therefore, we believe that focusing research on m^6^A-related ncRNAs may provide new opportunities for the identification of various molecular biomarkers and for the development of targeted drugs. Recent studies have shown that m^6^A-related lncRNAs can be used as prognostic biomarkers in lower-grade gliomas and lung adenocarcinomas [47]. However, studies based on the exploration of the molecular mechanisms and clinical applications of m^6^A-related ncRNAs in BC are few. Therefore, we attempted to use bioinformatics methods to construct the first m^6^A-related ncRNA–based prognostic risk model for BC.

In this study, we identified 183 m^6^A-related ncRNAs, of which 14 were associated with OS in BC. Finally, a prognostic risk assessment model consisting of 10 m^6^A-related ncRNAs was constructed. This model showed good prognostic evaluation performance in both the training and testing sets, and multivariate Cox regression analysis results revealed that the risk model could be deemed an independent prognostic factor for OS. The nomogram integrated independent risk factors that showed adequate prognostic evaluation performance and perfect consistency between the observed and predicted rates for the 1-year-, 3-year-, and 5-year OS. The above results demonstrated that the m^6^A-related ncRNA–based risk model had excellent performance in predicting patient outcomes and can be used as a potential prognostic biomarker. With its aid, we can identify high-risk patients early and implement targeted prevention through more active surveillance and aggressive management. For example, in NMIBC patients, we can increase the frequency of cystoscopy and extend the duration of bladder intravesical instillation. In addition, for MIBC patients, we can expand the scope of lymph node dissection and combine chemotherapy and radiotherapy after surgery. The advent of immune checkpoint inhibitors (ICIs) has revolutionized the treatment of BC, thereby ushering hope of a cure to patients with advanced BC [2]. However, only 20–30% of the patients with advanced BC will have long-lasting clinical benefits when subjected to treatment with ICIs [48]. Screening for potential beneficiaries of BC immunotherapy is an effective means of improving disease prognosis and achieving personalized medicine for BC. Of note, the risk model could be used to predict the outcomes of immunotherapy. Patients in the low-risk group were more likely to benefit from immunotherapy. Based on this model, we can preliminarily screen out patients who may benefit from immunotherapy, thereby reducing the economic burden on patients and society. Simultaneously, personalized medicine for BC patients can be achieved. Therefore, it is apparent that m^6^A-related ncRNAs are promising molecular biomarkers and can contribute to PPPM for BC.

Most m^6^A-related ncRNAs in risk models are closely related to cancer progression; for example, lncRNA HCP5 promotes human BC cell invasion and migration by targeting miR-29b-3p [49]; lncRNA GATA2-AS1 has been identified as a colon adenocarcinoma-related lncRNA [50] and is also involved in the growth regulation of non-small cell lung cancer [51]; lncRNA BAALC-AS1 regulates the proliferation of esophageal squamous cells by participating in the lncRNA BAALC-AS1/G3BP2/c-Myc feedback loop [52]; another study indicates that miR191 is abnormally expressed in more than 20 different malignancies [53], and that overexpressed miR191 in NMIBC can cause a significant decrease in EGR1 levels [54]. However, apart from lncRNA HCP5, much remains unknown regarding the underlying molecular mechanisms of the other 9 m^6^A-related ncRNAs involved in the progression of BC, and further studies are warranted.

We observed that the 1058 DEGs between the high- and low-risk groups were mainly enriched in molecular network systems associated with cellular immune responses (such as the chemokine-mediated signaling pathway, cellular response to interleukin-1, neutrophil chemotaxis, monocyte chemotaxis, and lymphocyte chemotaxis), and cellular metabolism (e.g., drug and xenobiotic metabolism by cytochrome P450, and steroid metabolism). On the one hand, the enrichment results explained and highlighted, to a certain extent, the potential applicability of the risk model in the prediction of immunotherapeutic responsiveness. On the other hand, they indicated that the biological processes related to the cellular immune response and biological metabolism should be the focus of future research to identify the role played by ncRNAs and their interactions established with m^6^A-related genes in BC.

This study presents with certain limitations. For example, although we used a testing set to evaluate the risk score model, there were fewer samples in this testing set, and our study was a retrospective study. In order to solve this limitation, we have reached agreements with multiple urology research centers in Guangdong Province to collect BC patient clinical information and sequencing data to build our own database, which can be used as a validation data set to evaluate research results based on public databases. Additionally, the specific role of m^6^A-related ncRNAs in BC risk models remains unclear, and further research should be conducted. We hope that through the publication of this paper, more researchers can pay attention to m^6^A-related ncRNAs and contribute to revealing the role of m^6^A-related ncRNAs in the occurrence and development of BC.

## Conclusions and expert recommendations

In conclusion, our study is the first to explore the potential application value of m^6^A-related ncRNAs in BC. We showed that the m^6^A-related ncRNA–based risk model demonstrated excellent performance in determining patient prognosis and immunotherapeutic responsiveness. Additionally, this model can aid in providing patients with targeted prevention and personalized medical services, thus projecting a new direction to improve PPPM for BC.

### Expert recommendations and outlook

The importance of sequencing technology for life science research is self-evident. At present, genomic and transcriptomic analysis of tumor tissues obtained by intraoperative resection and puncture biopsy has become a routine diagnosis and treatment of a variety of tumors. Previous studies based on sequencing data had paid more attention to various ncRNAs and m^6^A regulators, which had been proved to play an important regulatory role in carcinogenesis. This study is the first to explore the potential application value of m^6^A-related ncRNAs in BC, and we recommend this article to emphasize the importance of m^6^A-related ncRNAs in the basic and translational research for PPPM in BC.

Sequencing data for m^6^A-related ncRNAs analysis is not limited to tumor tissues sources. For urinary system tumors, especially BC, we think that exfoliated tumor cells (ETCs) in urine could provide a new direction for the study of m^6^A-related ncRNAs in BC. The research based on ETCs has the advantages of absolute non-invasive and high patient compliance, and shows a broad clinical application prospect.

## Supplementary Information

ESM 1 (DOCX 16 kb)

ESM 2 (XLSX 376 kb)

ESM 3 (XLSX 3816 kb)

ESM 4 (XLSX 133 kb)

ESM 5 (PNG 188 kb)

High resolution image (TIF 2828 kb)

ESM 6 (PNG 801 kb)

High resolution image (TIF 3333 kb)

ESM 7 (PNG 1470 kb)

High resolution image (TIF 4113 kb)

ESM 8 (PNG 263 kb)

High resolution image (TIF 1692 kb)

## References

[1] Bray F, Ferlay J, Soerjomataram I, Siegel RL, Torre LA, Jemal A (2018). Global cancer statistics 2018: GLOBOCAN estimates of incidence and mortality worldwide for 36 cancers in 185 countries. CA Cancer J Clin..

[2] Patel VG, Oh WK, Galsky MD (2020). Treatment of muscle-invasive and advanced bladder cancer in 2020. CA Cancer J Clin..

[3] Kamat AM, Hahn NM, Efstathiou JA, Lerner SP, Malmström P-U, Choi W (2016). Bladder cancer. Lancet..

[4] Akhtar M, Al-Bozom IA, Ben Gashir M, Taha NM (2019). Intrinsic molecular subclassification of urothelial carcinoma of the bladder: are we finally there?. Adv Anat Pathol..

[5] Cheng T, Zhan X (2017). Pattern recognition for predictive, preventive, and personalized medicine in cancer. EPMA J..

[6] Lu M, Zhan X (2018). The crucial role of multiomic approach in cancer research and clinically relevant outcomes. EPMA J..

[7] Pietzak EJ, Bagrodia A, Cha EK, Drill EN, Iyer G, Isharwal S (2017). Next-generation sequencing of nonmuscle invasive bladder cancer reveals potential biomarkers and rational therapeutic targets. Eur Urol..

[8] Miyamoto DT, Mouw KW, Feng FY, Shipley WU, Efstathiou JA (2018). Molecular biomarkers in bladder preservation therapy for muscle-invasive bladder cancer. Lancet Oncol..

[9] Fu Y, Dominissini D, Rechavi G, He C (2014). Gene expression regulation mediated through reversible m 6 A RNA methylation. Nat Rev Genet..

[10] Deng X, Su R, Weng H, Huang H, Li Z, Chen J (2018). RNA N 6-methyladenosine modification in cancers: current status and perspectives. Cell Res..

[11] Chen X-Y, Zhang J, Zhu J-S (2019). The role of m 6 A RNA methylation in human cancer. Mol cancer..

[12] Liu N, Pan T (2016). N 6-methyladenosine–encoded epitranscriptomics. Nat Struct Mol Biol..

[13] Roundtree IA, Evans ME, Pan T, He C (2017). Dynamic RNA modifications in gene expression regulation. Cell..

[14] Jia G, Fu Y, He C (2013). Reversible RNA adenosine methylation in biological regulation. Trends Genet..

[15] Ping X-L, Sun B-F, Wang L, Xiao W, Yang X, Wang W-J (2014). Mammalian WTAP is a regulatory subunit of the RNA N6-methyladenosine methyltransferase. Cell Res..

[16] Lan Q, Liu PY, Haase J, Bell JL, Hüttelmaier S, Liu T (2019). The critical role of RNA m6A methylation in cancer. Cell Res..

[17] Nachtergaele S, He C (2018). Chemical modifications in the life of an mRNA transcript. Annu Rev Genet..

[18] Lee M, Kim B, Kim VN (2014). Emerging roles of RNA modification: m6A and U-tail. Cell..

[19] Du J, Hou K, Mi S, Ji H, Ma S, Ba Y (2020). Malignant evaluation and clinical prognostic values of m6A RNA methylation regulators in glioblastoma. Front Oncol..

[20] Dai F, Wu Y, Lu Y, An C, Zheng X, Dai L (2020). Crosstalk between RNA m6A modification and non-coding RNA contributes to cancer growth and progression. Mol Ther Nucleic Acids..

[21] Beermann J, Piccoli M-T, Viereck J, Thum T (2016). Non-coding RNAs in development and disease: background, mechanisms, and therapeutic approaches. Physiol Rev..

[22] Esteller M (2011). Non-coding RNAs in human disease. Nat Rev Genet..

[23] Matsui M, Corey DR (2017). Non-coding RNAs as drug targets. Nat Rev Drug Discov..

[24] Li Y, Li G, Guo X, Yao H, Wang G, Li C (2020). Non-coding RNA in bladder cancer. Cancer Lett..

[25] Yi Y-C, Chen X-Y, Zhang J, Zhu J-S (2020). Novel insights into the interplay between m 6 A modification and noncoding RNAs in cancer. Mol Cancer..

[26] Ni W, Yao S, Zhou Y, Liu Y, Huang P, Zhou A (2019). Long noncoding RNA GAS5 inhibits progression of colorectal cancer by interacting with and triggering YAP phosphorylation and degradation and is negatively regulated by the m 6 A reader YTHDF3. Mol Cancer..

[27] Han J, Wang J-z, Yang X, Yu H, Zhou R, Lu H-C (2019). METTL3 promote tumor proliferation of bladder cancer by accelerating pri-miR221/222 maturation in m6A-dependent manner. Mol Cancer..

[28] Mariathasan S, Turley SJ, Nickles D, Castiglioni A, Yuen K, Wang Y (2018). TGFβ attenuates tumour response to PD-L1 blockade by contributing to exclusion of T cells. Nature..

[29] Tu Z, Wu L, Wang P, Hu Q, Tao C, Li K (2020). N6-Methylandenosine-related lncRNAs are potential biomarkers for predicting the overall survival of lower-grade glioma patients. Front Cell Dev Biol.

[30] Xu F, Huang X, Li Y, Chen Y, Lin L (2021). m6A-related lncRNAs are potential biomarkers for predicting prognoses and immune responses in patients with LUAD. Mol Ther Nucleic Acids..

[31] Friedman J, Hastie T, Tibshirani R (2010). Regularization paths for generalized linear models via coordinate descent. J Stat Softw..

[32] Robin X, Turck N, Hainard A, Tiberti N, Lisacek F, Sanchez J-C (2011). pROC: an open-source package for R and S+ to analyze and compare ROC curves. BMC Bioinformatics.

[33] Anders S, Huber W. Differential expression analysis for sequence count data. Nat Prec. 2010:1–12. 10.1186/gb-2010-11-10-r106 PMC3218662 20979621

[34] Lu M, Chen W, Zhuang W, Zhan X (2020). Label-free quantitative identification of abnormally ubiquitinated proteins as useful biomarkers for human lung squamous cell carcinomas. EPMA J..

[35] Thompson IM, Pauler DK, Goodman PJ, Tangen CM, Lucia MS, Parnes HL (2004). Prevalence of prostate cancer among men with a prostate-specific antigen level≤ 4.0 ng per milliliter. N Engl J Med..

[36] Ma S, Chan KW, Hu L, Lee TKW, Wo JYH, Ng IOL (2007). Identification and characterization of tumorigenic liver cancer stem/progenitor cells. Gastroenterol.

[37] Paez JG, Jänne PA, Lee JC, Tracy S, Greulich H, Gabriel S (2004). EGFR mutations in lung cancer: correlation with clinical response to gefitinib therapy. Science.

[38] Bolenz C, Lotan Y (2008). Molecular biomarkers for urothelial carcinoma of the bladder: challenges in clinical use. Nat Clin Pract Urol..

[39] Tan WS, Tan WP, Tan M-Y, Khetrapal P, Dong L, deWinter P (2018). Novel urinary biomarkers for the detection of bladder cancer: a systematic review. Cancer Treat Rev..

[40] Gorin MA, Verdone JE, Van Der Toom E, Bivalacqua TJ, Allaf ME, Pienta KJ (2017). Circulating tumour cells as biomarkers of prostate, bladder, and kidney cancer. Nat Rev Urol..

[41] Zhang S, Du L, Wang L, Jiang X, Zhan Y, Li J (2019). Evaluation of serum exosomal Lnc RNA-based biomarker panel for diagnosis and recurrence prediction of bladder cancer. J Cell Mol Med..

[42] Zhao X, Cui L (2019). Development and validation of a m6A RNA methylation regulators-based signature for predicting the prognosis of head and neck squamous cell carcinoma. Am J Cancer Res..

[43] Zhang J, Bai R, Li M, Ye H, Wu C, Wang C (2019). Excessive miR-25-3p maturation via N 6-methyladenosine stimulated by cigarette smoke promotes pancreatic cancer progression. Nat Commun..

[44] Zheng Z-Q, Li Z-X, Zhou G-Q, Lin L, Zhang L-L, Lv J-W (2019). Long noncoding RNA FAM225A promotes nasopharyngeal carcinoma tumorigenesis and metastasis by acting as ceRNA to sponge miR-590-3p/miR-1275 and upregulate ITGB3. Cancer Res..

[45] Shen X, Ling X, Lu H, Zhou C, Zhang J, Yu Q (2018). Low expression of microRNA-1266 promotes colorectal cancer progression via targeting FTO. Eur Rev Med Pharmacol Sci..

[46] Wang X, Zhang J, Wang Y (2019). Long noncoding RNA GAS5-AS1 suppresses growth and metastasis of cervical cancer by increasing GAS5 stability. Am. J Transl Res..

[47] Xu F, Huang X, Li Y, Chen Y, Lin L (2021). N6-Methyladenosine-related lncRNAs are potential biomarkers for predicting prognoses and immune responses in patients with lung adenocarcinoma. Mol Ther Nucleic Acids..

[48] Soares A, Carmo R, Rodrigues C, Grilo IT, Grande E (2020). Chemotherapy plus immune check-point inhibitors in metastatic bladder cancer. Bladder Cancer..

[49] Zhao C, Li Y, Hu X, Wang R, He W, Wang L (2020). LncRNA HCP5 promotes cell invasion and migration by sponging miR-29b-3p in human bladder cancer. OncoTargets Ther..

[50] Li Z, Tan H, Yu H, Deng Z, Zhou X, Wang M (2020). DNA methylation and gene expression profiles characterize epigenetic regulation of lncRNAs in colon adenocarcinoma. J Cell Biochem..

[51] Zhang L, Gao L, Shao M, Sun G (2019). A MYC target long non-coding RNA GATA2-AS1 regulates non-small cell lung cancer growth. Neoplasma..

[52] Zhang H, Wang Y, Zhang W, Wu Q, Fan J, Zhan Q (2021). BAALC-AS1/G3BP2/c-Myc feedback loop promotes cell proliferation in esophageal squamous cell carcinoma. Cancer Commun..

[53] Nagpal N, Kulshreshtha R (2014). miR-191: an emerging player in disease biology. Front Genet..

[54] Lee J-Y, Yun SJ, Jeong P, Piao X-M, Kim Y-H, Kim J (2018). Identification of differentially expressed miRNAs and miRNA-targeted genes in bladder cancer. Oncotarget..

