# Supplementary material for: N6-methyladenosine-related non-coding RNAs are potential prognostic and immunotherapeutic responsiveness biomarkers for bladder cancer
Source: EPMA J. 2021 Oct 21;12(4):589–604. doi: 10.1007/s13167-021-00259-w (PMC8648947; doi:10.1007/s13167-021-00259-w)
Supplement: Supplementary file 1 — (DOCX 16 kb) [file 13167_2021_259_MOESM1_ESM.docx]

**Supplementary materials**

**Supplementary Table 1** The correlation analysis of m^6^A regulators and miRNAs in TCGA.

**Supplementary Table 2** The correlation analysis of m^6^A regulators and lncRNAs in TCGA.

**Supplementary Table 3** 1058 differentially expressed genes (DEGs) and annotation information.

**Supplementary Fig. 1** Clinical information of 408 tumor samples in TCGA.

**Supplementary Fig. 2** The expression pattern of 14 OS related ncRNAs between tumor and normal tissues in TCGA. 6 ncRNAs (MIR324, MIR25, AL022311.1, AC012615.1, AC026362.1, and GATA2-AS1) were significantly increased in tumor tissues.

**Supplementary Fig. 3** There was significant difference in gene expression pattern between the high- and low-risk groups in TCGA. **a.** The distribution of 1058 differentially expressed genes (DEGs) between the high-and low-risk groups in TCGA. **b.** Gene expression pattern in the high- and low-risk groups.

**Supplementary Fig. 4** 12 OS related ncRNAs were significantly differential expressed between the high- and low-risk groups in TCGA. Except for HCP5, the other ncRNAs are all low expressed in the high-risk group).
