# Supplementary figures and images for: N6-methyladenosine-related non-coding RNAs are potential prognostic and immunotherapeutic responsiveness biomarkers for bladder cancer
Source: EPMA J. 2021 Oct 21;12(4):589–604. doi: 10.1007/s13167-021-00259-w (PMC8648947; doi:10.1007/s13167-021-00259-w)

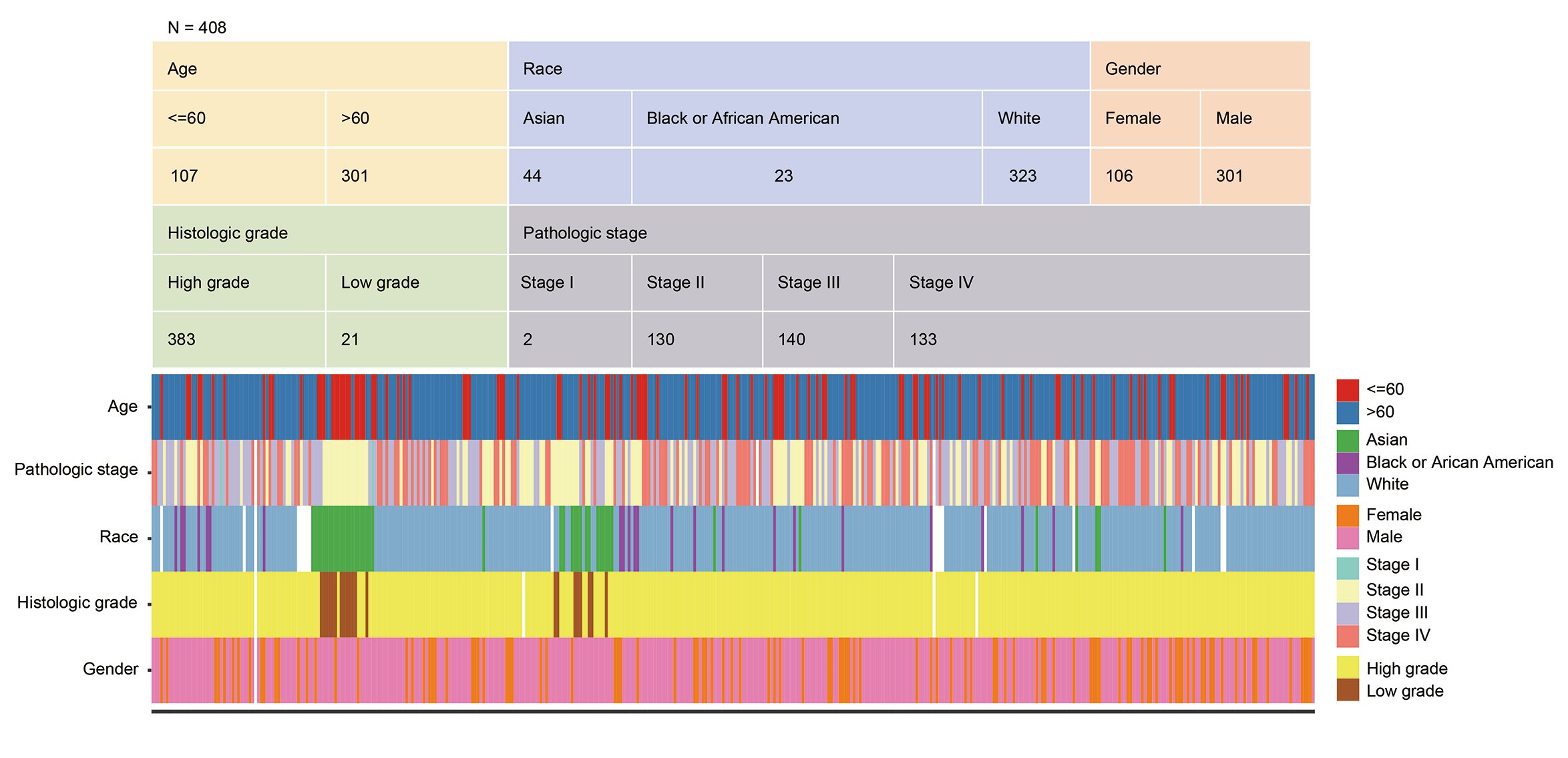

Supplement: Supplementary file 5 — (PNG 188 kb) [file 13167_2021_259_Fig9_ESM.png]

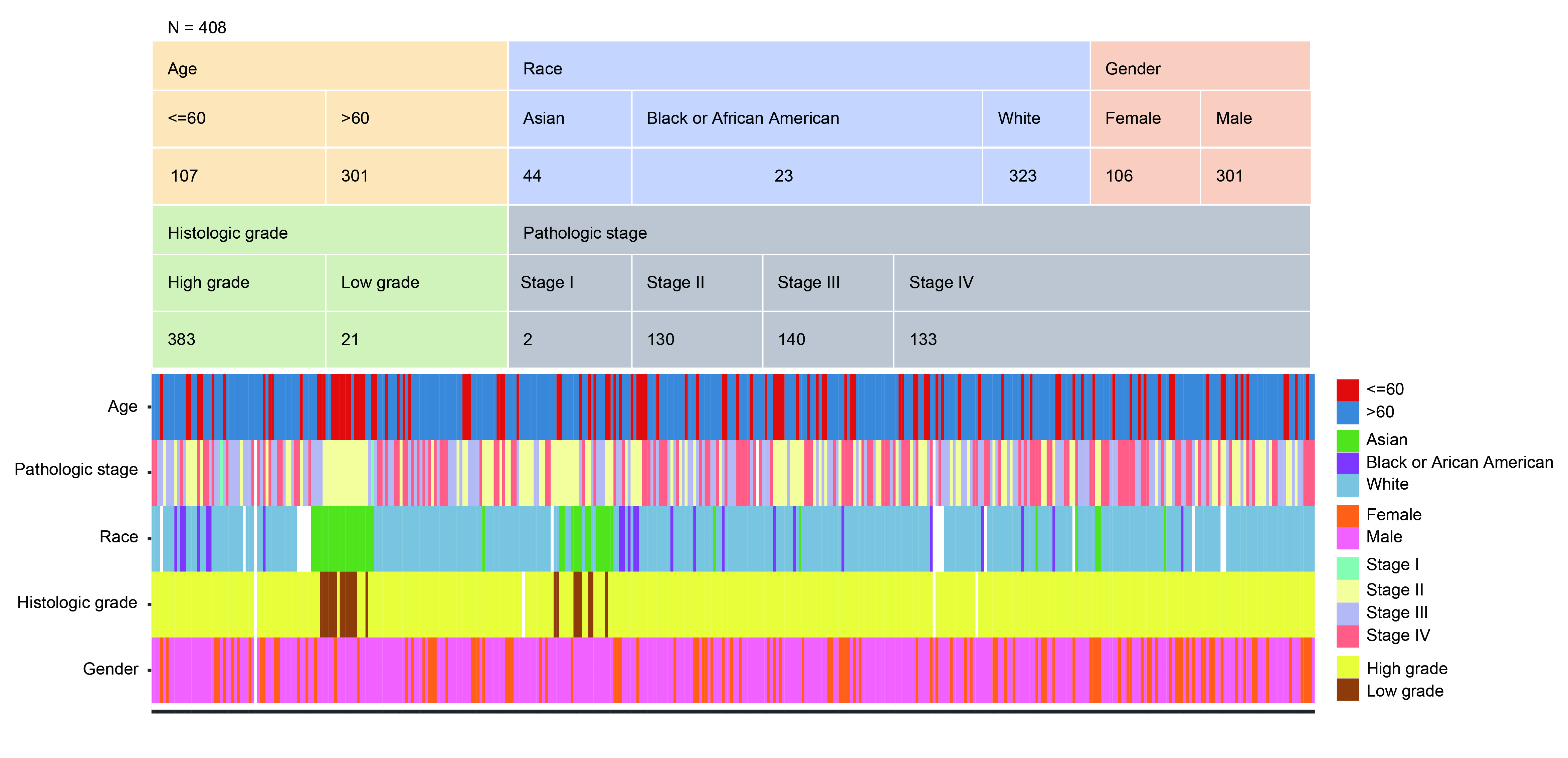

Supplement: Supplementary file 6 — High resolution image (TIF 2828 kb) [file 13167_2021_259_MOESM5_ESM.tif]

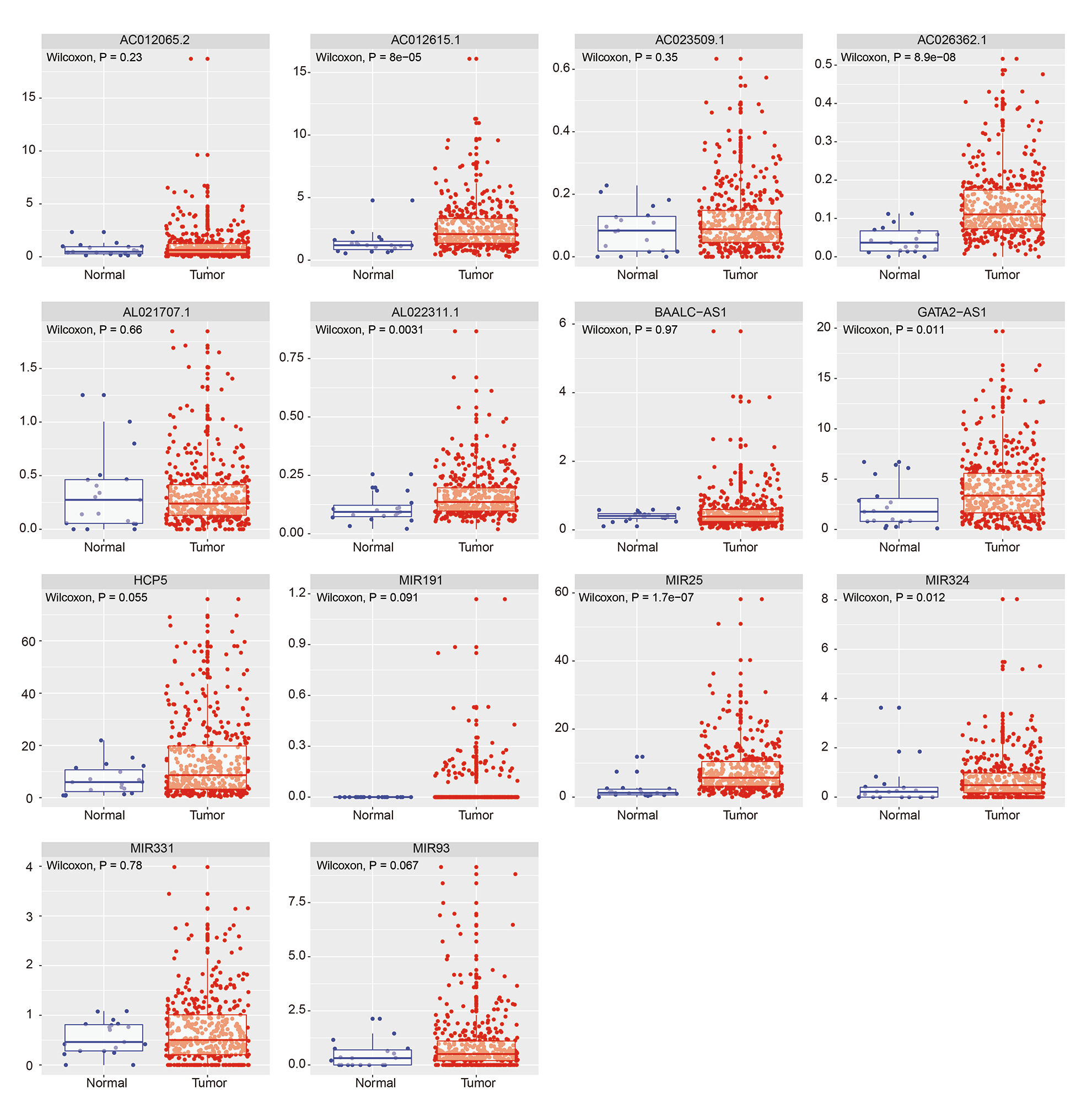

Supplement: Supplementary file 7 — (PNG 801 kb) [file 13167_2021_259_Fig10_ESM.png]

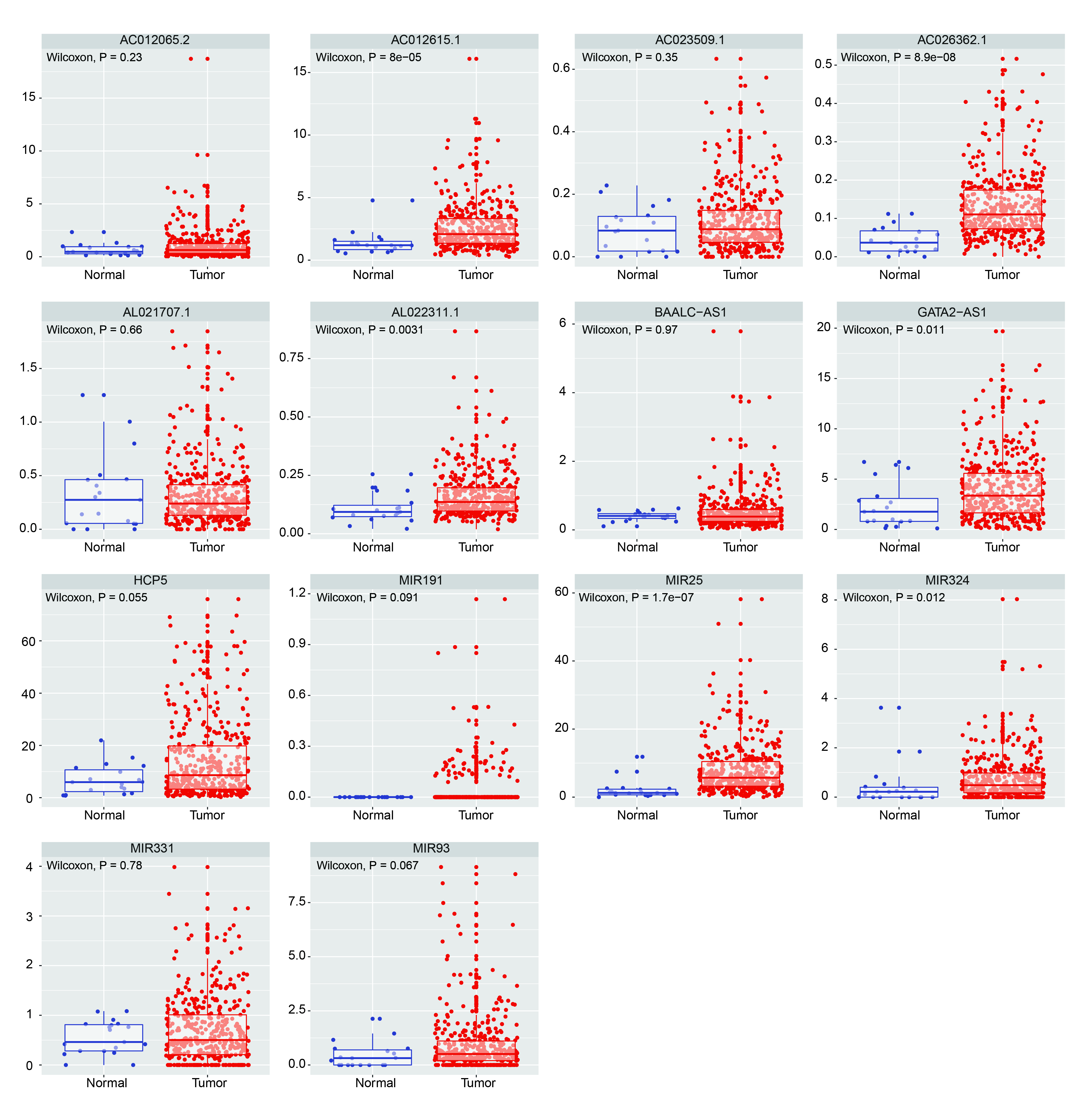

Supplement: Supplementary file 8 — High resolution image (TIF 3333 kb) [file 13167_2021_259_MOESM6_ESM.tif]

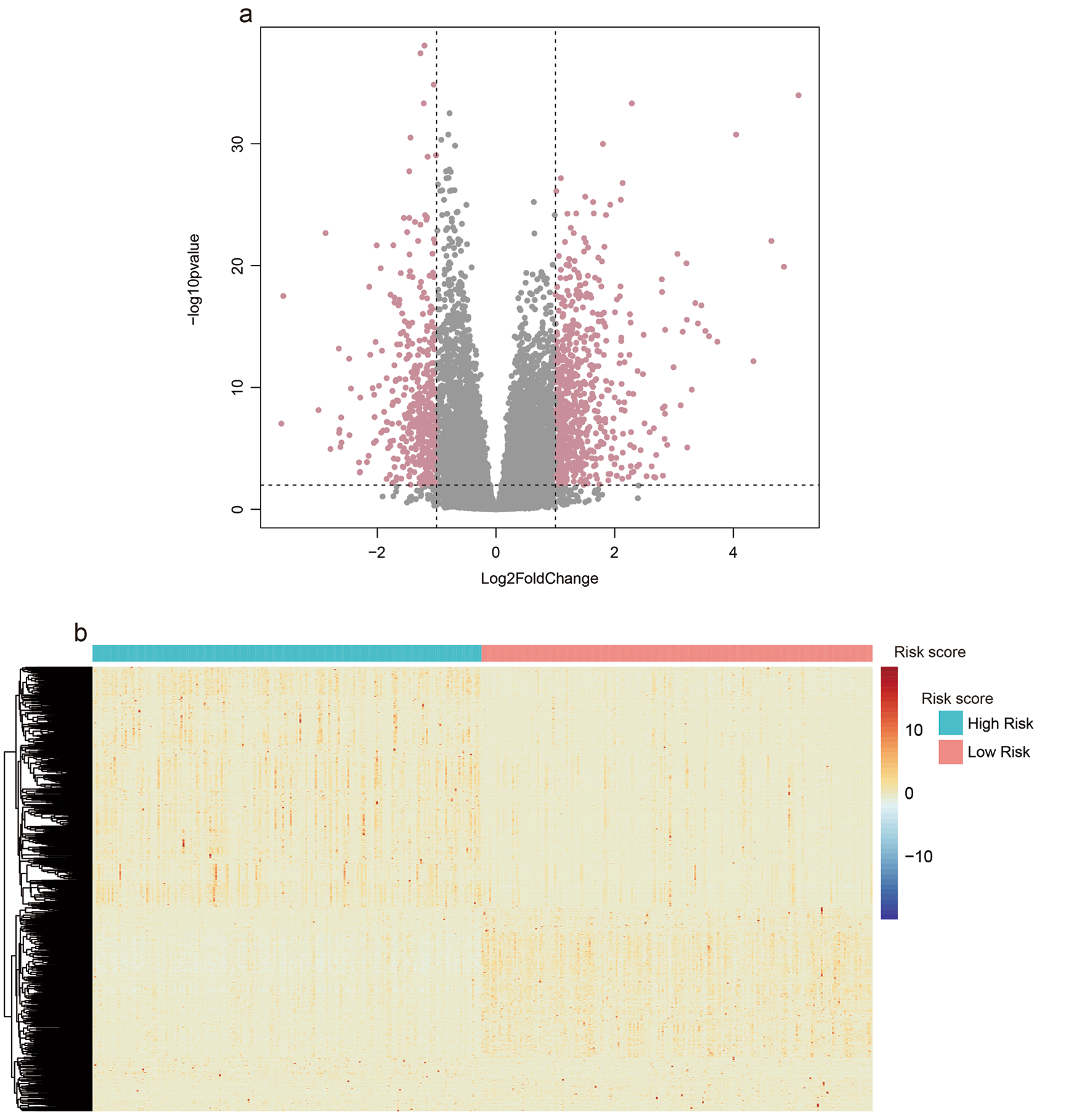

Supplement: Supplementary file 9 — (PNG 1470 kb) [file 13167_2021_259_Fig11_ESM.png]

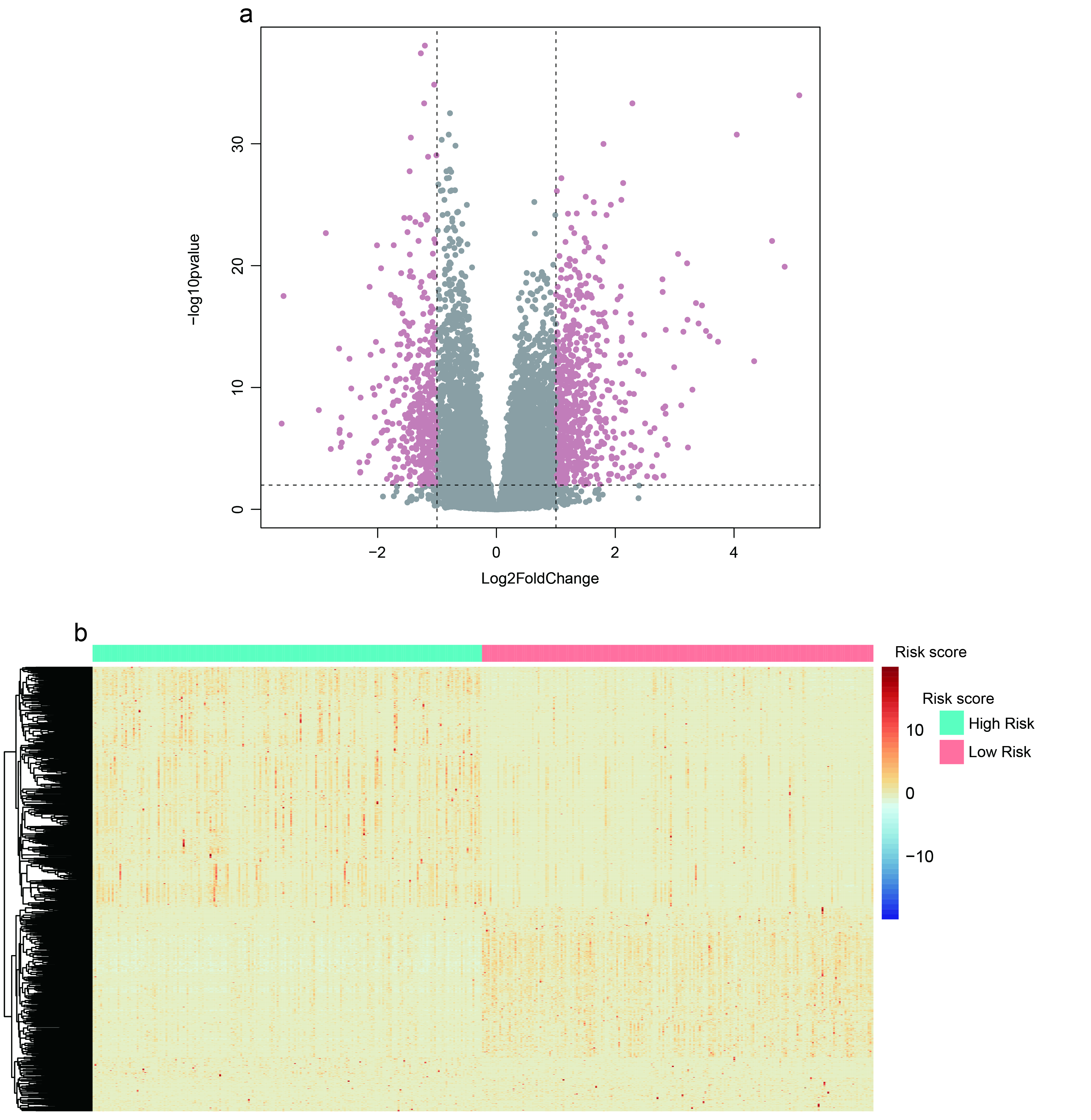

Supplement: Supplementary file 10 — High resolution image (TIF 4113 kb) [file 13167_2021_259_MOESM7_ESM.tif]

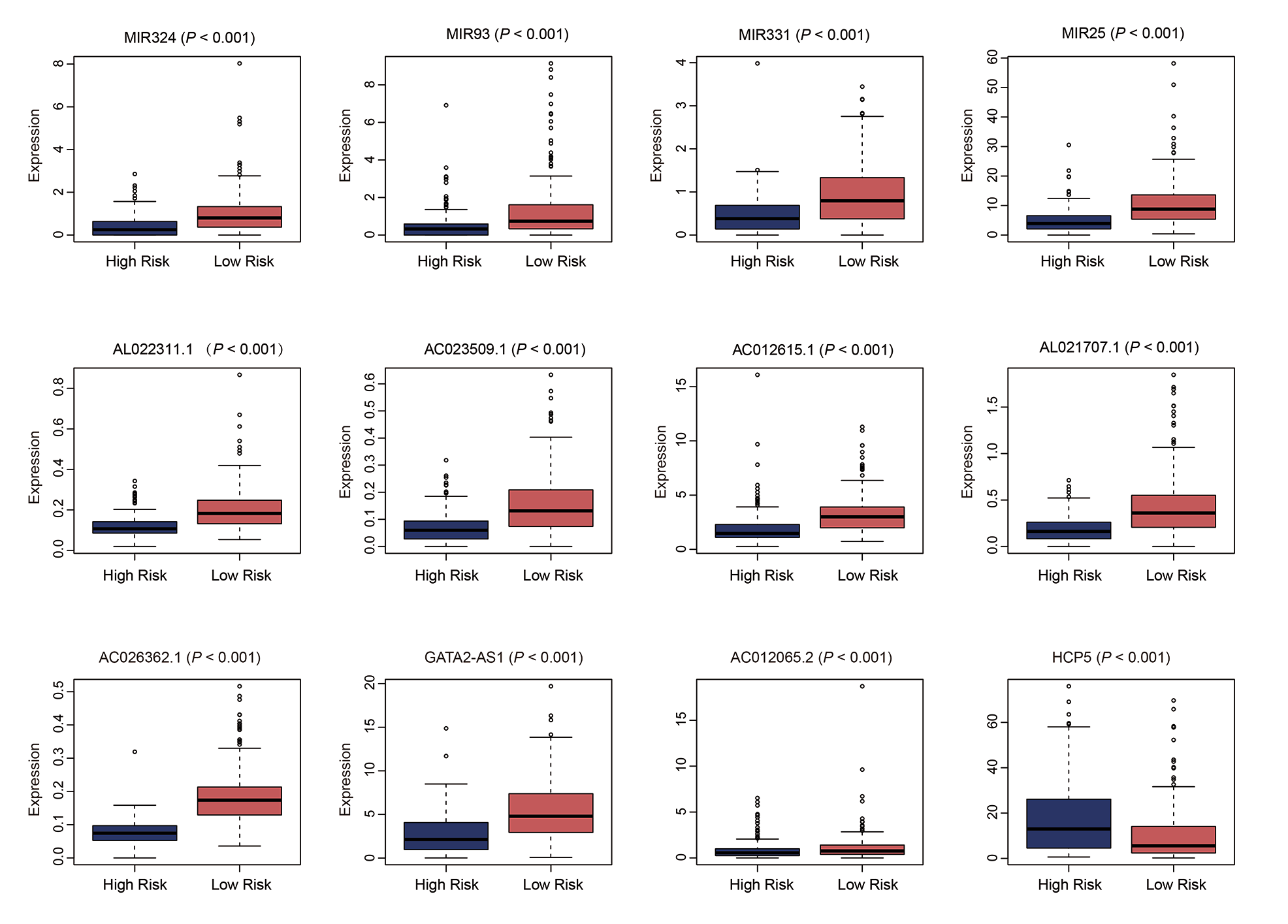

Supplement: Supplementary file 11 — (PNG 263 kb) [file 13167_2021_259_Fig12_ESM.png]

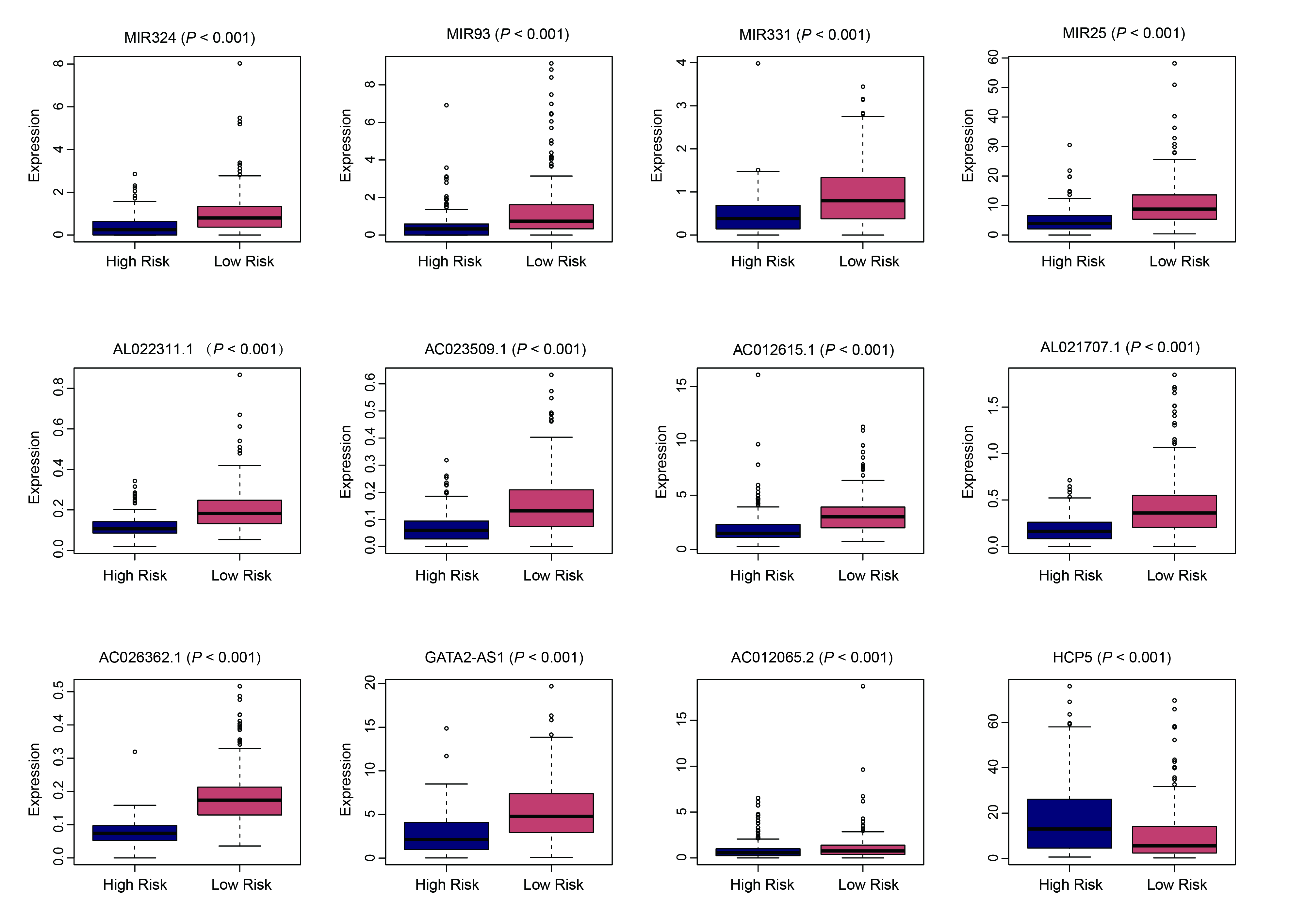

Supplement: Supplementary file 12 — High resolution image (TIF 1692 kb) [file 13167_2021_259_MOESM8_ESM.tif]
